# Intermittent Drying With Heated Air and Microwaves of Dragon Fruit Foams Formulated With Different Protein Sources

**DOI:** 10.1111/1750-3841.71460

**Published:** 2026-09-13

**Authors:** Matheus de Souza Cruz, Amanda Aparecida De Lima Santos, Gabriela Fonsêca Leal, Jhenifer Cristina Carvalho Santos, Guilherme Mathias Lopes, Leandro Levate Macedo, Irineu Petri Júnior, Jefferson Luiz Gomes Corrêa

**Affiliations:** ^1^ Department of Food Science Federal University of Lavras Lavras MG Brazil; ^2^ Department of Agricultural Engineering Federal University of Lavras Lavras MG Brazil; ^3^ Faculty of Engineering Federal University of Grande Dourados Dourados MS Brazil; ^4^ Department of Chemical Engineering Federal University of Lavras Lavras MG Brazil

**Keywords:** albumin, foam mat drying, pea protein, pitaya, soy protein, whey protein

## Abstract

This study aimed to investigate red dragon fruit powder production by foam mat drying, combining a simplex centroid mixture design for foam formulation with intermittent convective drying and intermittent microwave drying. Albumin, pea protein, and soy protein were evaluated as alternative foaming agents to whey protein, and optimization was based on density, overrun, and porosity, while drainage stability was used as a qualitative screening parameter. The optimized pea protein foam and whey protein control were dried by both intermittent methods at 40°C and 60°C. Drying kinetics, powder properties, color, protein content, phenolics, flavonoids, betalains, antioxidant activity, and energy indicators were evaluated. Pea protein provided the best balance among foam responses, producing low‐density foams (0.14 g/mL), with 470.75% overrun and 0.82 porosity. Intermittent convective drying required up to 233 min at 40°C and 127 min at 60°C, whereas intermittent microwave drying achieved maximum drying time reductions of 65.67% and 78.74%, respectively. Under laboratory conditions, intermittent microwave drying increased the specific moisture extraction rate (from 0.0286 to 0.3764 kg/kWh) and reduced the specific energy consumption (from 31.4380 to 3.5805 kWh/kg). All powders showed water activity below 0.50. Drying at 40°C favored bioactive retention, with total phenolics from 177.83 to 216.80 mg GAE/100 g, betalains from 40.609 to 61.880 mg/100 g, DPPH from 56.190% to 64.242%, and ABTS from 55.745% to 62.478%. These findings indicate that pea protein showed comparable performance to whey protein under the tested conditions, while intermittent microwave drying provides a faster and more energy‐efficient route to obtain pitaya powders rich in bioactive compounds.

## 1 Introduction

Pitaya (*Hylocereus* spp., synonym *Selenicereus* spp.), also known as dragon fruit, stands out for its unique sensory attributes and for presenting a complex nutritional matrix composed of vitamins, minerals, dietary fiber, and phenolic compounds (Ferreira et al. 2023). Among these constituents, betalains deserve special attention because they are nitrogen‐containing, water‐soluble pigments responsible for the intense color of the peel and pulp. They are also associated with significant antioxidant activity and potential anti‐inflammatory effects (Martinez et al. 2024; Shah et al. 2023). Despite those characteristics, the high water activity and substantial sugar content of fresh fruit favor accelerated deterioration and senescence processes, highlighting the need for processing technologies capable of extending fruit shelf life (Wang et al. 2024).

Foam mat drying has been established as a suitable alternative for converting fruit pulps and extracts into powders, since air incorporation into the food matrix increases the exposed surface area, reduces resistance to water diffusion, and favors moisture removal (Macedo et al. 2021; Kumar et al. 2023; Çalışkan Koç et al. 2022). Foaming agents are particularly relevant in this context because they adsorb at the air–water interface, reduce interfacial tension, and form cohesive films around air bubbles (Kumar et al. 2023; Khatri et al. 2024). Whey protein is widely used because of its well‐established interfacial properties, whereas plant proteins, such as pea and soy proteins, are attractive alternatives for vegan and more sustainable formulations. Albumin also remains relevant because of its high foaming capacity and technological accessibility (Araújo et al. 2022; Chandrakar et al. 2024; Lopes et al. 2024; Vimercati et al. 2022; Brar et al. 2020; Kalambe and Guhe 2026).

Although foam porosity favors mass transfer, heat transfer during convective drying (CD) may still be limited by the discontinuous gas phase and the low effective thermal conductivity of the foam layer (Dehghannya and Habibi‐Ghods 2025). In this context, microwave drying represents a promising alternative for intensifying water removal, as electromagnetic energy promotes volumetric heating through dipole rotation and ionic migration, thereby generating internal vapor pressure and accelerating evaporation (Joardder and Karim 2025; Santos et al. 2025a). However, continuous microwave application may cause rapid temperature increases, non‐uniform heating, and localized overheating, particularly as moisture content decreases during drying (Silveira et al. 2024). Therefore, intermittent operation in both convective and microwave drying may favor heat and moisture redistribution during periods without energy input, thereby improving process control, potentially reducing energy demand and promoting better preservation of powder quality (Macedo et al. 2022; Santos et al. 2025b).

Recent studies have already demonstrated the applicability of foam mat drying to pitaya, including the optimization of foaming agent concentration and whipping time, the use of ethanol pretreatment, the evaluation of drying temperature and the assessment of foaming systems for improving powder quality and bioactive retention (Araújo et al. 2022; Macedo et al. 2021; Akman et al. 2024; Mulyadi et al. 2024; Rahayu et al. 2024; Yuwono et al. 2026; Firdhauzi et al. 2025). However, most studies have focused on isolated foaming agents, selected concentrations, or conventional drying strategies, with whey protein commonly used as a reference protein because of its well‐established foaming functionality. Thus, the use of a simplex centroid mixture design represents an important advance because it allows the individual and combined effects of alternative protein sources to be evaluated in the optimization of red pitaya foams. In addition, to the best of our knowledge, no studies have evaluated intermittent microwave drying applied to red pitaya foams or its direct comparison with intermittent CD.

The objective of this study was to investigate the production of red pitaya powder by foam mat drying in two complementary stages. In the first stage, red pitaya foams were formulated using albumin, pea protein, and soy protein, evaluated both individually and in blends according to a simplex centroid mixture design, as alternative foaming agents to whey protein. The foam formulation was optimized using the desirability function, considering density, overrun, and porosity as response variables, while drainage stability was evaluated as a qualitative screening parameter. In the second stage, the optimized foam and the whey protein control were subjected to intermittent CD and intermittent microwave drying at 40°C and 60°C. The resulting powders were evaluated for drying kinetics, energy performance, physical properties, color, protein content, phenolics, flavonoids, betalains, antioxidant activity, microstructure, and multivariate relationships.

## 2 Materials and Methods

### 2.1 Material

Pitayas (*Hylocereus polyrhizus*) with red peel and red pulp were purchased from a local producer in the region of Lavras, MG, Brazil (21°14′43″ S; 44°59′59″ W). Fruits were selected based on ripeness criteria (predominantly red peel), soluble solids content (12.56 ± 0.84 °Brix), and physical integrity (absence of lesions). After selection, the fruits were washed, sanitized in a sodium hypochlorite solution (200 mg/L for 15 min), and pulped. Pulps containing the seeds were packed in plastic bags and stored at –18°C until the experiments were performed. Thawing was carried out according to a methodology adapted from Santos et al. (2023). Initially, samples were refrigerated (4°C) for 12 h, then incubated at 25°C in a temperature‐controlled chamber (Eletrolab EL111/4, São Paulo, Brazil) for 1 h. Subsequently, they were homogenized using a mechanical stirrer, and the resulting extract was immediately used in the subsequent steps.

The food‐grade foaming agents used in this study were commercial whey protein concentrate, egg white albumin, pea protein isolate (NewNutrition, Ribeirão Preto, SP, Brazil), and soy protein isolate (Empório Terra Vitta, São Paulo, SP, Brazil). All protein powders were used as received. Xanthan gum (Êxodo Científica, Sumaré, SP, Brazil) was used as the stabilizer. The water solubility index (WSI) values, determined as described in Section 2.6.4, were 83.75% ± 1.78% for whey protein, 80.59% ± 1.23% for albumin, 79.49% ± 0.83% for pea protein, and 80.78% ± 2.48% for soy protein, with no significant differences among the evaluated protein sources (*p* > 0.05). The manufacturer‐declared nutritional compositions of the commercial protein powders are presented in Table S1 of the Supplementary Material.

### 2.2 Foam Preparation and Experimental Design

For foam preparation, 5% (w/w) of the foaming agent and 1% (w/w) of the stabilizing agent were added to pitaya extract (100 g). The mixture was initially homogenized in a blender (Britânia, Joinville, SC, Brazil) at maximum speed (level 4) for 1 min. Subsequently, the solution was transferred to a mixer (Mondial, Conceição do Jacuípe, Brazil) and whipped for 5 min, with the speed gradually increased during the first 30 s to the maximum level, resulting in foam formation.

The experimental design used a simplex‐centroid mixture design with three center‐point repetitions (Table 1), considering albumin, pea protein, and soy protein as mixture components. In addition, a control formulation was prepared exclusively with whey protein. This control formulation was not included in the simplex‐centroid design or in the response surface modeling but was used as an external reference to compare the optimized alternative‐protein foam with a conventional whey‐protein‐based foam during the drying stage. The independent variables evaluated were *X*_1_ = albumin, *X*_2_ = pea protein, and *X*_3_ = soy protein, tested as alternatives to whey protein. The fixed concentrations of foaming agent and xanthan gum were selected based on preliminary tests, which indicated that these were the lowest levels capable of producing foams suitable for characterization and drying, with adequate stability, lower density, higher overrun, and higher porosity. At lower concentrations, the systems showed insufficient air incorporation and poor structural stability, compromising foam formation and the subsequent drying assays. Therefore, the mixture design was used to evaluate the relative proportions of albumin, pea protein, and soy protein within the fixed total foaming‐agent concentration, rather than to optimize the total amount of foaming agent or stabilizer.

**TABLE 1:** Experimental formulations of the simplex‐centroid mixture design for evaluation of foaming agents and experimental results for foam properties.

Treatments	Coded values			Actual concentrations (%)			Foam properties			
	*X*_1_	*X*_2_	*X*_3_	*X*_1_	*X*_2_	*X*_3_	Density (g/mL)	Overrun (%)	Porosity (–)	Stability (%)
Control*	—	—	—	—	—	—	0.12 ± 0.01	484.66 ± 25.69	0.82 ± 0.01	100.00 ± 0.00
**T1**	1.00	0.00	0.00	5.00	0.00	0.00	0.15 ± 0.01	311.45 ± 16.89	0.76 ± 0.01	100.00 ± 0.00
**T2**	0.00	1.00	0.00	0.00	5.00	0.00	0.14 ± 0.02	470.75 ± 27.56	0.82 ± 0.01	100.00 ± 0.00
**T3**	0.00	0.00	1.00	0.00	0.00	5.00	0.30 ± 0.08	139.96 ± 37.23	0.63 ± 0.04	100.00 ± 0.00
**T4**	0.50	0.50	0.00	2.50	2.50	0.00	0.19 ± 0.00	279.81 ± 40.07	0.73 ± 0.03	100.00 ± 0.00
**T5**	0.50	0.00	0.50	2.50	0.00	2.50	0.18 ± 0.02	329.17 ± 26.51	0.77 ± 0.01	100.00 ± 0.00
**T6**	0.00	0.50	0.50	0.00	2.50	2.50	0.17 ± 0.00	393.42 ± 19.61	0.80 ± 0.01	100.00 ± 0.00
**T7**	0.66	0.17	0.17	3.30	0.85	0.85	0.18 ± 0.02	357.69 ± 28.85	0.78 ± 0.01	100.00 ± 0.00
**T8**	0.17	0.66	0.17	0.85	3.30	0.85	0.21 ± 0.02	285.65 ± 40.15	0.74 ± 0.03	100.00 ± 0.00
**T9**	0.17	0.17	0.66	0.85	0.85	3.30	0.25 ± 0.01	223.68 ± 44.85	0.69 ± 0.04	100.00 ± 0.00
**T10**	0.33	0.33	0.33	1.67	1.67	1.67	0.22 ± 0.00	287.06 ± 23.27	0.74 ± 0.02	100.00 ± 0.00
**T11**	0.33	0.33	0.33	1.67	1.67	1.67	0.21 ± 0.01	302.39 ± 33.79	0.75 ± 0.02	100.00 ± 0.00
**T12**	0.33	0.33	0.33	1.67	1.67	1.67	0.22 ± 0.00	298.25 ± 34.51	0.75 ± 0.02	100.00 ± 0.00

*Note: X*_1_ = albumin, *X*_2_ = pea protein and *X*_3_ = soy protein. (‐) = dimensionless parameter. The values are expressed as the mean of triplicates ± standard deviation (*n* = 3). *Formulated using exclusively whey protein as a foaming agent at a concentration of 5% (w/w). This formulation was not included in the simplex‐centroid mixture design or in the response surface modeling and was used only as an external reference.

The special cubic Scheffé polynomial model was fitted to the data related to foam properties, as presented in Equation (1), where *ŷ* is the estimated response; *β_i_*, *β*_ij_, and *β*_ij__k_ are the regression coefficients; and *X_i_*, *X_j_*, and *X_k_* correspond to the concentrations of albumin, pea protein, and soy protein, respectively. Analysis of variance (ANOVA) was used to evaluate the statistical significance of each response variable. The best models to represent each parameter were selected after removing non‐significant terms (Vimercati et al. 2022).

(1) $$\hat{y}=\sum_{i=1}^{3}\beta_{i}X_{i}+\sum \sum_{i\;<\;j}^{3}\beta_{ij}X_{i}X_{j}+\sum \sum \sum_{i\;<\;j\;<\;k}^{3}\beta_{ijk}X_{i}X_{j}X_{k}$$

### 2.3 Foam Properties and Optimization

Foams were evaluated for density, porosity, expansion, and stability, and all analyses were performed in triplicate. During the assays, the foam was handled with extreme care to avoid structural disruption or bubble collapse.

#### 2.3.1 Density

To determine density, foams were transferred to a 25 mL glass funnel and weighed on an analytical balance (Shimadzu, AUY220 model, Brazil). The value was calculated according to Equation (2) as the ratio between foam mass (*m*) and volume (*v*) (Nunes et al. 2022).

(2) $$\mathrm{Density}\;\left(g/\mathrm{mL}\right)=\frac{m}{v}$$

#### 2.3.2 Overrun

Overrun was calculated based on Equation (3), considering the mass of the extract with foaming agent (*M_i_*) and the mass of the foam (*M_f_*), obtained after completely filling the volume of a 25 mL glass funnel in both determinations (Araújo et al. 2022).

(3) $$\mathrm{Overrun}\;\left(\%\right)=\frac{M_{i}\;-M_{f}}{M_{f}}\times \;100$$

#### 2.3.3 Porosity

According to Macedo et al. (2021), porosity was calculated using the foam density (*ρ*_foam_) and the density of the extract with the foaming agent (*ρ*_extract_), as shown in Equation (4).

(4) $$\mathrm{Porosity}\;=1\;-\;\frac{\rho_{\;\mathrm{foam}}}{\rho_{\;\mathrm{extract}}}$$

#### 2.3.4 Stability

Foam stability was determined according to Hossain et al. (2024), with adaptations. Briefly, 75 mL of foam (*V_i_*) was placed in a Büchner funnel (55 mm in diameter), and the assembly was kept over a graduated cylinder inside a thermostatically controlled oven (Solab SL104/40, Piracicaba, Brazil) at 60°C for 240 min. The main adaptations consisted of increasing the assay temperature and extending the evaluation time to impose a more severe stability condition, corresponding to the highest drying temperature evaluated in this study. After this period, the volume of liquid drained from the foam (*V_d_*) was measured in mL. Foam stability was calculated as the non‐drained fraction of the initial foam volume, according to Equation (5).

(5) $$\mathrm{Stability}\;\left(\%\right)=\frac{V_{i}\;-\;V_{d}}{V_{i}}\;\times 100$$

where *V_i_* is the initial foam volume (mL), and *V_d_* is the drained liquid volume after 240 min (mL). When no liquid drainage was observed, *V_d_* was considered equal to zero, resulting in 100% foam stability.

#### 2.3.5 Optimization

The optimized foam formulation was selected using the desirability function in Statistica software, version 14.0.1.8 (StatSoft Inc., Tulsa, OK, USA). The optimization criteria were defined to minimize foam density and maximize overrun and porosity (Derringer and Suich 1980). Foam stability was used as a qualitative screening parameter to confirm that all foams were suitable for the subsequent drying assays.

Experimental validation was conducted under the operating conditions identified to achieve the values predicted by the desirability function for foam properties, aiming to verify the adequacy of the model equations in predicting the optimal responses. Agreement between predicted and experimental values was evaluated using the *χ* ^2^ goodness‐of‐fit test, as presented in Equation (6) (Lopes et al. 2024).

(6) $$\chi^{2}=\sum_{i=1}^{n}\frac{{\left(E-P\right)}^{2}}{P}$$

where *E* is the observed experimental value and *P* is the corresponding predicted value.

In addition, the experimental value obtained for the formulation corresponding to the optimum point was directly compared with the control foam to verify significant differences between means by ANOVA followed by Tukey's test (*p* < 0.05), using Statistica software, version 14.0.1.8 (StatSoft Inc., Tulsa, OK, USA).

### 2.4 Drying Preparation

The optimum foam formation condition and the control treatment, with initial moisture contents of 81.06% ± 0.60% and 81.28% ± 0.67%, respectively, were subjected to the drying conditions described in Table 2. Each run was carried out with 60.50 ± 0.26 g of sample, spread in a circular container 26 cm in diameter to form a foam layer 0.5 cm thick. During the process, the circular sample holder was rotated by 90° at each weighing to minimize the effect of tray position in the dryer. The drying kinetics were obtained by periodic weighing: every 5 min during the first hour of processing and thereafter every 10 min until the samples reached a final moisture content of approximately 8.00% (wet basis), ensuring water activity below 0.6 and microbiological stability. The mass of the samples was determined on an analytical balance (Marte Científica, AD33000 model, Brazil) with an accuracy of ±0.01 g.

**TABLE 2:** Experimental design for foam drying.

Codes	Foam	Drying method	Temperature (°C)
C‐CD40	Control	Convective drying	40
C‐CD60	Control	Convective drying	60
T2‐CD40	T2	Convective drying	40
T2‐CD60	T2	Convective drying	60
C‐MW40	Control	Microwave drying	40
C‐MW60	Control	Microwave drying	60
T2‐MW40	T2	Microwave drying	40
T2‐MW60	T2	Microwave drying	60

*Note*: Treatment codes: *C* = control foam (whey protein concentrate); T2 = optimized foam (pea protein); CD = convective drying; MW = microwave drying; 40 and 60 = drying air temperatures (°C).

After drying, samples were packed in sealed laminated packages, protected from light and oxygen, and subsequently stored in a desiccator at room temperature or in a domestic freezer at –18°C until analyses were performed (Macedo et al. 2022).

#### 2.4.1 Intermittent Microwave Drying

Microwave drying was carried out in an adapted domestic microwave oven (Brastemp BMG45, 32 L, 900 W) coupled to a digital controller that allowed manual adjustment of the reference temperature (setpoint). The actual microwave power was determined as 732.7 ± 34.5 W using the IMPI 2 L method (Buffler 1993). The equipment was operated at 100% of its available power throughout the drying process, corresponding to an operational power density of approximately 12.21 W/g.

Temperature was continuously monitored using a type‐*K* thermocouple inserted into the foam layer. The thermocouple was introduced through an access opening located in the upper central region of the standard sample holder used in the microwave oven. Its sensing junction was positioned at the geometric center of the 26‐cm‐diameter circular container, corresponding to 13 cm from its edge, and remained fixed throughout each drying run. The thermocouple assembly was electrically grounded to reduce microwave‐induced electromagnetic interference and improve operational safety. Potential temperature non‐uniformity was minimized by maintaining a uniform foam thickness, rotating the sample holder by 90° at each weighing interval, and operating the magnetron intermittently under thermocouple‐based on–off control. To characterize the thermal profile, the minimum, mean, and maximum temperatures recorded during intermittent microwave drying are presented in Supplementary Material Table S2, representing the actual temperature variation observed for each treatment.

When intermittent microwave drying was performed, the intermittent regime was controlled automatically using an on–off control based on the thermocouple signal. In this mechanism, the magnetron operated intermittently until the target temperature was reached, then automatically switched off, turning on again when the sample temperature dropped below the setpoint, maintaining the process close to the established value. The duty cycle was calculated as the ratio between the magnetron‐on time and the total drying time. When expressed as equivalent 1 min operating cycles, the magnetron remained active for approximately 9.0 s and inactive for 51.0 s at 40°C. At 60°C, the magnetron remained active for approximately 23.0 s and inactive for 37.0 s. Operating time was controlled by the conventional oven panel and defined based on periodic mass readings throughout the process.

#### 2.4.2 Intermittent Convective Drying

The convective treatment was performed in an experimental system developed by the research group, consisting of a drying chamber (53 × 32.5 × 45 cm). Air supply was provided by a radial fan (200 m^3^/h) connected to the drying chamber by a galvanized steel duct (56 × 34 × 24 cm; 1 mm thickness) insulated with glass wool (5 cm). Inside the duct, electrical resistances were positioned perpendicular to the airflow, promoting heating. The inlet of heated air into the chamber passed through an opening (10 × 8 cm) located on the rear wall. A second orifice (10 × 8 cm) located on the side wall allowed hot‐air exhaust (Junqueira et al. 2017).

The convective dryer operated at 110 V, with an estimated nominal resistance power of 1370 W and an average system power consumption of approximately 600 W. The air velocity was 2.2 m/s, previously calibrated using a digital anemometer (Incoterm, Germany). Air temperature was continuously monitored by a thermocouple, and the resistance was activated to heat the samples until the set temperature was reached, switching off automatically when this limit was attained. When the temperature decreased below the setpoint, the system was automatically activated again. This on/off mechanism maintained the temperature close to the setpoint throughout the process. The duty cycle was calculated as the ratio between the resistance‐on time and the total drying time. When expressed as equivalent 1 min operating cycles, the electrical resistance remained active for approximately 17.0 s and inactive for 43.0 s at 40°C, while the corresponding active and inactive periods at 60°C were approximately 36.0 and 24.0 s, respectively.

To monitor the thermal profile, the minimum, mean, and maximum temperatures recorded during CD are presented in Supplementary Material Table S2, representing the real temperature variation observed for each treatment.

#### 2.4.3 Moisture Ratio and Drying Rate

Moisture ratio (MR) was calculated from the relationship between moisture content at a given time (*X_t_*), initial moisture content (*X*_0_), and equilibrium moisture content (*X_e_*), according to Equation (7). Equilibrium was reached at a moisture content of 7.59% ± 1.27% on a wet basis (0.0821 kg water/kg dry matter), as no further measurable mass variation was observed in successive measurements under the applied drying conditions. Drying rate (DR) was determined from the reduction in moisture content between two consecutive time intervals and the corresponding time difference, as presented in Equation (8) (Cruz et al. 2025a).

(7) $$\mathrm{MR}=\frac{X_{t}\;-\;X_{e}}{X_{0}\;-\;X_{e}}$$

(8) $$\mathrm{DR}=\frac{X_{t}\;-\;X_{t+\Delta t}}{\Delta t}\;$$

where MR is the dimensionless moisture ratio; *X_t_* is the moisture content at drying time *t* (kg water/kg dry matter); *X*_0_ is the initial moisture content (kg water/kg dry matter); *X_e_* is the equilibrium moisture content under the applied drying conditions (kg water/kg dry matter); DR is the drying rate (kg water/kg dry matter/min); *X_t_*_+_Δ*_t_* is the moisture content at the subsequent drying time (kg water/kg dry matter); and Δ*t* is the time interval between two consecutive measurements (min).

#### 2.4.4 Apparent Effective Moisture Diffusivity

The apparent effective moisture diffusivity coefficient (*D*_eff_) was estimated using the analytical solution of Fick's second law for an infinite slab geometry, according to Crank (1975). The model assumes uniform initial moisture distribution, constant moisture diffusivity and temperature, negligible external resistance, and negligible shrinkage. In this study, because the foams were spread as a thin layer, the drying process was considered as thin‐layer drying, and the slab geometry was adopted. The general solution is presented in Equation (9).

(9) $$\mathrm{MR}\;=\left[\frac{8}{\pi^{2}}\;\sum_{i=0}^{\infty }\frac{1}{{\left(2i+1\right)}^{2}}\;\exp\;\left(-{\left(2i+1\right)}^{2}\pi^{2}\;D_{\mathrm{eff}}\frac{t}{4L^{2}}\right)\right]\;$$

where MR is the dimensionless moisture ratio, *D*_e__ff_ is the apparent effective moisture diffusivity coefficient (m^2^/s), *t* is the drying time (s), *i* is the term index of the infinite series, and *L* is the characteristic length, corresponding to half of the foam layer thickness (m).

For sufficiently long drying periods, only the first term of the series was considered, corresponding to *i* = 0. Therefore, the apparent *D*_e__ff_ was calculated from the slope of the linear regression of ln(MR) as a function of drying time, according to the simplified relationship ln(MR) = ln(8/*π* ^2^)−(*π* ^2^ *D*_e__ff_/4*L* ^2^)*t*. Thus, *D*_e__ff_ was obtained from the slope of the fitted line. The obtained values were therefore interpreted as apparent effective moisture diffusivity coefficients rather than true intrinsic diffusivities. This distinction is necessary because the analytical Fickian solution does not explicitly account for possible shrinkage of the foam layer, time‐dependent changes in diffusivity, or internal temperature gradients. These limitations are particularly relevant for microwave drying, in which electromagnetic energy absorption may generate non‐uniform heating and localized temperature differences within the sample. Therefore, apparent *D*_eff_ values were interpreted as comparative drying indices among treatments under a consistent geometric assumption, rather than as true intrinsic moisture diffusivities.

The parameters of the drying models were estimated by nonlinear regression using Statistica software, version 14.0.1.8 (StatSoft Inc., Tulsa, OK, USA), through least‐squares minimization of the residuals between experimental and predicted MR values (Santos et al. 2025c). The criteria used to evaluate goodness of fit were the coefficient of determination (*R* ^2^) (Equation (10)), reduced chi‐square (*χ* ^2^) (Equation (11)), and root mean square error (RMSE) (Equation (12)).

(10) $$R^{2\;}=1\;-\;\left[\frac{\sum_{i=1}^{N}{\left(MR_{\exp,i}\;-\;MR_{\mathrm{pre},i}\right)}^{2}}{\sum_{i=1}^{N}{\left(MR_{\exp,i}\;-\;MR_{\exp,\mathrm{mean}}\right)}^{2}}\right]\;$$

(11) $$\chi^{2}=\frac{\sum_{i=1}^{N}{\left(MR_{\exp,i}\;-\;MR_{\mathrm{pre},i}\right)}^{2}}{N\;-\;n}\;$$

(12) $$\mathrm{RMSE}\;=\sqrt{\frac{\sum_{i=1}^{N}{\left[MR_{\exp,i}\;-\;MR_{\mathrm{pre},i}\right]}^{2}}{N}}\;$$

where *R* ^2^ is the coefficient of determination; *χ* ^2^ is the reduced chi‐square; RMSE is the root mean square error; MR_exp,_ *_i_* is the experimental moisture ratio at the *i*‐th observation; MR_pre,_ *_i_* is the predicted moisture ratio at the *i*‐th observation; MR_exp, mean_ is the mean experimental moisture ratio; *N* is the number of experimental observations; and *n* is the number of constants in the mathematical model.

#### 2.4.5 Energy Consumption

To evaluate the energy consumption associated with the drying processes, a digital energy meter (Moko Smart, MK114B model, China) was connected to the power supply of each drying system. The equipment recorded the total electrical energy consumption in kWh, including the dryer and auxiliary components required for process operation. Therefore, the measured value corresponded to the total energy consumption of the drying system under the experimental conditions used. Energy consumption was measured in three independent drying runs for each treatment.

The recorded energy values were used to calculate the specific moisture extraction rate (SMER) and specific energy consumption (SEC) of the drying process, according to Equations (13) and (14), respectively (Tepe 2025). Both parameters were calculated using the same experimental quantities, namely the total mass of water removed during drying and the total electrical energy consumed during the drying process. SMER expresses the amount of water removed per unit of energy consumed, whereas SEC expresses the amount of energy required to remove one unit of water.

(13) $$\mathrm{SMER}\;=\frac{m_{v}}{E_{t}}\;$$

(14) $$\mathrm{SEC}\;=\frac{E_{t}}{m_{v}}\;$$

where SMER is the specific moisture extraction rate (kg water/kWh), SEC is the specific energy consumption (kWh/kg water), *m_v_* is the total mass of water removed during drying (kg water), and *E_t_* is the total electrical energy consumed during drying (kWh).

### 2.5 Microstructural Analysis

Scanning electron microscopy (SEM) was used to analyze pitaya powders. Samples were placed on aluminum stubs and fixed onto an aluminum foil film using carbon double‐sided adhesive tape, properly identified. Samples were then coated with a thin carbon layer using a carbon evaporator (Bal‐Tec). Coated samples were visualized in a scanning electron microscope (Tescan, CLARA FEG‐SEM model, Libušina, Czech Republic) operating at 20 kV and a working distance of 10 mm.

### 2.6 Characterization of Dried Samples

#### 2.6.1 Yield

The yield of the dried powders was calculated on a dry matter basis, considering the relationship between the dry mass of the powder obtained after drying and the initial dry matter of the foam formulation. The initial dry matter included the solids from the pitaya extract and the dry matter of the additives added to the formulation, including the foaming agent and xanthan gum. The dry mass of the powder was corrected according to its final moisture content. Yield was calculated according to Equation (15) (Costa et al. 2025).

(15) $$\mathrm{Yield}\;\;\left(\%\right)=\frac{m_{p}\times \;TS_{p}}{\left(m_{e}\times \;TS_{e}\right)+\;DM_{a}}\;\;\times \;100$$

where *m_p_* is the mass of powder obtained after drying (g); TS*_p_* is the total solids fraction of the dried powder; *m_e_* is the mass of pitaya extract used in the foam formulation (g); TS*_e_* is the total solids fraction of the pitaya extract; and DM*_a_* is the total dry matter of the additives added to the formulation, including the foaming agent and xanthan gum (g).

#### 2.6.2 Bulk and Tapped Density

Bulk density was calculated by measuring the volume occupied by 2 g of powder in a 10 mL graduated cylinder without compaction. Tapped density, in turn, was determined by tapping the powder‐filled cylinder (10 taps onto a fabric mat from a height of 15 cm) before recording the final volume. Bulk and tapped densities were calculated using Equations (16) and (17), respectively (Yüksel 2021).

(16) $$\mathrm{Bulk}\;\mathrm{density}\;(\rho_{B})=\frac{\mathrm{Mass}\;\mathrm{of}\;\mathrm{powder}\;\left(g\right)}{\mathrm{Volume}\;\mathrm{of}\;\mathrm{powder}\;\left(cm^{3}\right)}\;$$

(17) $$\mathrm{Tap}\;\mathrm{density}\;(\rho_{T})=\frac{\mathrm{Mass}\;\mathrm{of}\;\mathrm{powder}\;\left(g\right)}{\mathrm{Final}\;\mathrm{tapped}\;\mathrm{volume}\;\left(cm^{3}\right)}\;$$

#### 2.6.3 Flowability and Cohesiveness

Sample flowability and cohesiveness were evaluated using the Carr index (CI) (Equation (18)) and Hausner ratio (HR) (Equation (19)), both derived from bulk and tapped densities (Salgado‐Aristizabal et al. 2025). Flowability classification based on CI and HR values is presented in Supplementary Material Table S3.

(18) $$\mathrm{Carr}\;\mathrm{index}\;(\%)=\frac{\rho_{T}\;-\;\rho_{B}}{\rho_{T}}\;\times \;100\;$$

(19) $$\mathrm{Hausner}\;\mathrm{ratio}=\frac{\rho_{T}\;}{\rho_{B}}\;$$

#### 2.6.4 Water Solubility Index (WSI) and Water Absorption Index (WAI)

A 0.5 g sample of dried pitaya powder was added to 10 mL of distilled water. The mixture was stirred at room temperature for 30 min and then centrifuged at 2260 rpm for 15 min (Vimercati et al. 2022). After centrifugation, the supernatant was transferred to a plate and dried in an oven until complete water removal. The WSI was calculated as the percentage ratio between the mass of dissolved solids recovered from the dried supernatant and the initial dry mass of the sample, according to Equation (20). The water absorption index (WAI) was calculated as the ratio between the mass of wet sediment retained after centrifugation and the initial dry mass of the sample, according to Equation (21) (Bogusz et al. 2024).

(20) $$\mathrm{WSI}\;\left(\%\right)=\frac{\mathrm{mass}\;\mathrm{of}\;\mathrm{dissolved}\;\mathrm{solids}\;\mathrm{in}\;\mathrm{the}\;\mathrm{supernatant}\;}{\mathrm{mass}\;\mathrm{of}\;\mathrm{dry}\;\mathrm{sample}}\;\times \;100$$

(21) $$\mathrm{WAI}\;\left(g/g\right)=\frac{\mathrm{mass}\;\mathrm{of}\;\mathrm{wet}\;\mathrm{sediment}\;}{\mathrm{mass}\;\mathrm{of}\;\mathrm{dry}\;\mathrm{sample}}\;$$

#### 2.6.5 Hygroscopicity

Hygroscopicity was determined according to the methodology described by Milani et al. (2024). Approximately 1 g of sample was placed in Petri dishes inside a desiccator containing a saturated NaCl solution, providing approximately 75% relative humidity at the assay temperature. After this period, samples were weighed again to evaluate water absorption. Hygroscopicity was expressed as grams of water absorbed per gram of dry sample.

#### 2.6.6 Wettability

Wettability was determined according to the method of Vimercati et al. (2022). Briefly, 100 mL of distilled water at 25°C was placed in a 250 mL beaker, and 0.1 g of the sample was carefully added to the water through a funnel. The time required for all powder particles to penetrate the water was recorded using a digital stopwatch. Results were expressed in seconds and used to compare sample wettability.

#### 2.6.7 Moisture Content

Sample moisture content was determined gravimetrically in a vacuum oven (Solab SL104/40, Piracicaba, Brazil) at 70°C until constant weight (AOAC 2023).

#### 2.6.8 Water Activity

Water activity was determined using a hygrometer (Aqualab, 3‐TE model, Washington, USA) at 25°C.

### 2.7 Color Parameters

Sample color was determined using a portable colorimeter (LS173 model; Shenzhen Linshang Technology Co., Ltd., Shenzhen, China) using the CIELAB system. The instrument was previously calibrated using the white standard provided by the manufacturer. Analyses were performed at room temperature, obtaining L* (lightness), a* (green‐to‐red coordinate), and b* (blue‐to‐yellow coordinate). Chroma (C*) and hue angle (*h°*) were calculated according to Equations (22) and (23), respectively (Cruz et al. 2025b).

(22) $$C^{*}=\sqrt{{\left(a^{*}\right)}^{2}+{\left(b^{*}\right)}^{2}}\;$$

(23) $$h^{\circ }=\;\mathrm{ta}n^{-1}\;\left(\frac{b*}{a*}\right)\;\times \;\frac{180}{\pi }\;$$

where C* represents color saturation; *h*° represents the hue angle, expressed in degrees; and a* and b* are the color coordinates of the dried powder samples.

### 2.8 Protein Content

Total protein content was determined by the Kjeldahl method according to AOAC (2023). The procedure consisted of three steps: digestion, distillation, and titration. The final protein content was obtained by multiplying the nitrogen content by the nitrogen‐to‐protein conversion factor of 6.25.

### 2.9 Bioactive Compounds Analysis

Extract preparation for the determination of bioactive compounds was performed according to the method of Zitha et al. (2022). Briefly, 2 g of previously macerated dried pitaya powder was mixed with 20 mL of aqueous methanol (80%, v/v) and then subjected to ultrasound‐assisted extraction for 10 min at room temperature. After 20 min of stirring in the dark at room temperature, the extracts were filtered through filter paper, and the residues were extracted once more under the same conditions described above. The two extracts were combined, and bioactive compound analyses were performed in triplicate using a microplate reader (Biochrom EZ Read 2000).

#### 2.9.1 Total Phenolic Compounds

Total phenolic content was determined using adaptations of the Fast Blue method described by Medina (2011). Initially, a 0.2 mL aliquot of the extract was transferred to borosilicate tubes, and 0.8 mL of distilled water, 0.1 mL of 0.1% Fast Blue BB solution, and 0.1 mL of 5% NaOH solution were added sequentially. Samples were then shaken for 1 min and kept in the dark for 90 min. After this period, 300 µL aliquots were transferred to microtitration plates. Absorbance was read at 420 nm using a microplate reader.

Quantification was performed using a calibration curve prepared with gallic acid standard solutions subjected to the same reaction conditions as the samples. A gallic acid stock solution (1 mg/mL) and working standard solutions at concentrations of 2.5, 5.0, 7.5, 10.0, 15.0, 20.0, and 25.0 µg/mL were prepared in deionized water. A 1 mL aliquot of each standard solution was transferred to borosilicate tubes, followed by the addition of 0.1 mL of 0.1% Fast Blue BB reagent. The mixture was shaken for 1 min, and then 0.1 mL of 5% NaOH solution was added. The reaction was conducted at room temperature for 90 min. After this period, 300 µL aliquots were transferred to microtitration plates, and optical density was measured at 420 nm. Gallic acid concentrations were plotted as a function of optical density, and the calibration curve was fitted by linear regression. The final calculations considered the dry matter content of the samples, the initial volume of the extracts, and all dilution factors applied to the extracts before analysis. The values obtained in µg of gallic acid equivalents were converted to mg and expressed as mg GAE per 100 g of sample on a dry basis.

#### 2.9.2 Total Flavonoid Content

Total flavonoid content was determined using a direct spectrophotometric method based on the conversion coefficient described by Francis (1982) and Leal et al. (2025). The extracts were centrifuged, and the supernatant was collected for analysis. Absorbance was measured at 380 nm using a spectrophotometer, with the extraction solution used as the blank. No external calibration curve with catechin, quercetin, or rutin was used. Instead, absorbance values were directly converted using the method‐specific conversion coefficient for flavonoids (CA = 766). Results were expressed as milligrams of total flavonoids per 100 g of sample on a dry basis, according to Equation (24).

(24) $$\mathrm{Total}\;\mathrm{flavonoid}\;\mathrm{content}\;\left(\mathrm{mg}\cdot \;100\;g^{-1}\right)=\frac{A\;\times \;\mathrm{FD}\;\times \;1000}{m\;\times \;\mathrm{CA}}\;$$

where *A* is absorbance; FD is the dilution factor (volume/mass); *m* is sample mass in grams; and CA is the conversion coefficient for flavonoids, adopted as 766.

#### 2.9.3 Betalains Determination

Betalain content was determined according to the method described by Qingzhu et al. (2016). After centrifugation, the supernatant was collected, and absorbance was measured at 538 and 483 nm using a spectrophotometer, with the extraction solution used as the blank. Concentrations of betacyanins (*λ* = 538 nm) and betaxanthins (*λ* = 483 nm) were calculated by Equations (25) and (26), respectively. Total betalain content was obtained by summing the betacyanin and betaxanthin fractions. The results were expressed as mg of total betalain per 100 g of sample on a dry basis.

(25) $$\mathrm{Betacyanin}\;\left(\mathrm{mg}\cdot \;100\;g^{-1}\right)=\frac{A_{538\;}\times \;\mathrm{DF}\;\times \;\mathrm{MM}\;\times \;V\;\times \;100}{\varepsilon \;\times \;P\;\times \;l}\;$$

(26) $$\mathrm{Betaxanthin}\;\left(\mathrm{mg}\cdot \;100\;g^{-1}\right)=\frac{A_{483}\times \;\mathrm{DF}\;\times \;\mathrm{MM}\;\times \;V\;\times \;100}{\varepsilon \;\times \;P\;\times \;l}\;$$

where *A*_538_ is the absorbance for betacyanins measured at 538 nm; *A*_483_ is the absorbance for betaxanthins measured at 483 nm; DF is the dilution factor; MM is the molecular mass (550 g·mol^−1^ for betanin and 308 g·mol^−1^ for indicaxanthin); V is the volume of the pigment solution (mL); *ε* is the molar extinction coefficient (60,000 L·mol^−1^·cm^−1^ for betanin and 48,000 L·mol^−1^·cm^−1^ for indicaxanthin); *l* is the path length (1 cm); and *P* is the sample mass used for extraction, corrected to dry matter (*g*).

#### 2.9.4 Antioxidant Potential

Antioxidant activity was determined using the ABTS•+ and DPPH assays. For the ABTS•+ assay, the radical cation was generated by reacting a 7 mM ABTS solution with 2.45 mM potassium persulfate and maintaining the mixture in the dark for 16 h. The resulting ABTS•+ solution was diluted with ethanol to obtain an absorbance of 0.70 ± 0.02 at 734 nm. Subsequently, 30 µL of the sample extract was added to 300 µL of the diluted ABTS•+ solution. The control reaction was prepared by replacing the sample extract with 30 µL of the extraction solvent while maintaining the same volume of the ABTS•+ solution. After 6 min of reaction, absorbance was measured at 734 nm using ethanol as the spectrophotometric blank. The procedure was performed according to Re et al. (1999), with adaptations. In the DPPH assay, 10 µL of extract was added to 290 µL of DPPH solution, incubated in the dark for 30 min, and the absorbance was recorded at 515 nm in a spectrophotometer with methanol as the blank. For the DPPH assay, the control reaction was prepared using 10 µL of the extraction solution and 290 µL of the DPPH solution (Cheng et al. 2006). Results of both analyses were expressed as free‐radical scavenging percentage (%FRS), as established in Equation (27).

(27) $$\begin{array}{ccc} & & \%\;\mathrm{FRS}\;=\\ & & \frac{\mathrm{Absorbance}\;\mathrm{of}\;\mathrm{the}\;\mathrm{control}\;-\;\mathrm{Absorbance}\;\mathrm{of}\;\mathrm{the}\;\mathrm{sample}}{\mathrm{Absorbance}\;\mathrm{of}\;\mathrm{the}\;\mathrm{control}}\;\times \;100\; \end{array}$$

### 2.10 Statistical Analysis

ANOVA was performed to evaluate the experimental data, and mean comparisons were carried out using Tukey's test when significant effects were observed. The significance level adopted for all tests was 0.05. Statistical analyses were conducted using Statistica software, version 14.0.1.8 (StatSoft Inc., Tulsa, OK, USA).

In the first experimental stage, foam formulations were evaluated using a simplex‐centroid mixture design with albumin, pea protein, and soy protein as mixture components. Density, overrun, porosity, and foam stability were determined in triplicate and expressed as mean ± standard deviation. The data were fitted using a Scheffé mixture model, and response surfaces were analyzed to evaluate the effects of the protein sources and their interactions on foam properties. The optimized formulation was selected by the desirability function, aiming to minimize density and maximize overrun and porosity. Foam stability was used only as a qualitative screening criterion. The selected formulation was then experimentally validated by comparing the predicted and experimental values. The whey protein control formulation was not included in the mixture design modeling and was used only as an external reference for comparison with the optimized formulation selected by the desirability function.

In the second experimental stage, the optimized foam and the whey protein control foam were dried using a completely randomized 2 × 2 × 2 factorial design. The factors were drying method (intermittent CD and intermittent microwave drying), drying temperature (40°C and 60°C), and foam formulation (optimized foam and whey protein control). Thus, the main effects of drying method, drying temperature, and foam formulation, as well as their two‐way and three‐way interaction effects, were evaluated using factorial ANOVA/GLM. The responses analyzed in the factorial stage included drying kinetics, duty cycle, energy consumption, SEC, SMER, apparent effective moisture diffusivity, model‐fitting parameters, physical properties, color parameters, protein content, bioactive compounds, and antioxidant activity of the powders. Quantitative results were expressed as mean ± standard deviation. When significant effects were observed, the means of the relevant factor levels or treatment combinations were compared using Tukey's test at *p* < 0.05. Before ANOVA, the assumptions of normality and homogeneity of variance were checked using the Shapiro–Wilk and Levene tests, respectively.

Additionally, principal component analysis (PCA) was performed to investigate the relationships between the drying treatments and the quantitative sample‐characterization variables. Before PCA, the variables were mean‐centered and scaled to unit variance using *z*‐score standardization because they were expressed in different units and numerical ranges. Accordingly, PCA was performed using the correlation matrix. The treatments and variables were projected onto the factorial plane defined by Factors 1 and 2. The contribution of each variable was interpreted from its loading magnitude and direction, while the treatment scores were used to identify associations among drying method, temperature, foam formulation, and powder characteristics. For interpretative purposes, variables with absolute loading values equal to or greater than 0.70 were considered strong contributors to the corresponding factor.

## 3 Results and Discussion

### 3.1 Foam Properties and Optimization

**FIGURE 1:**
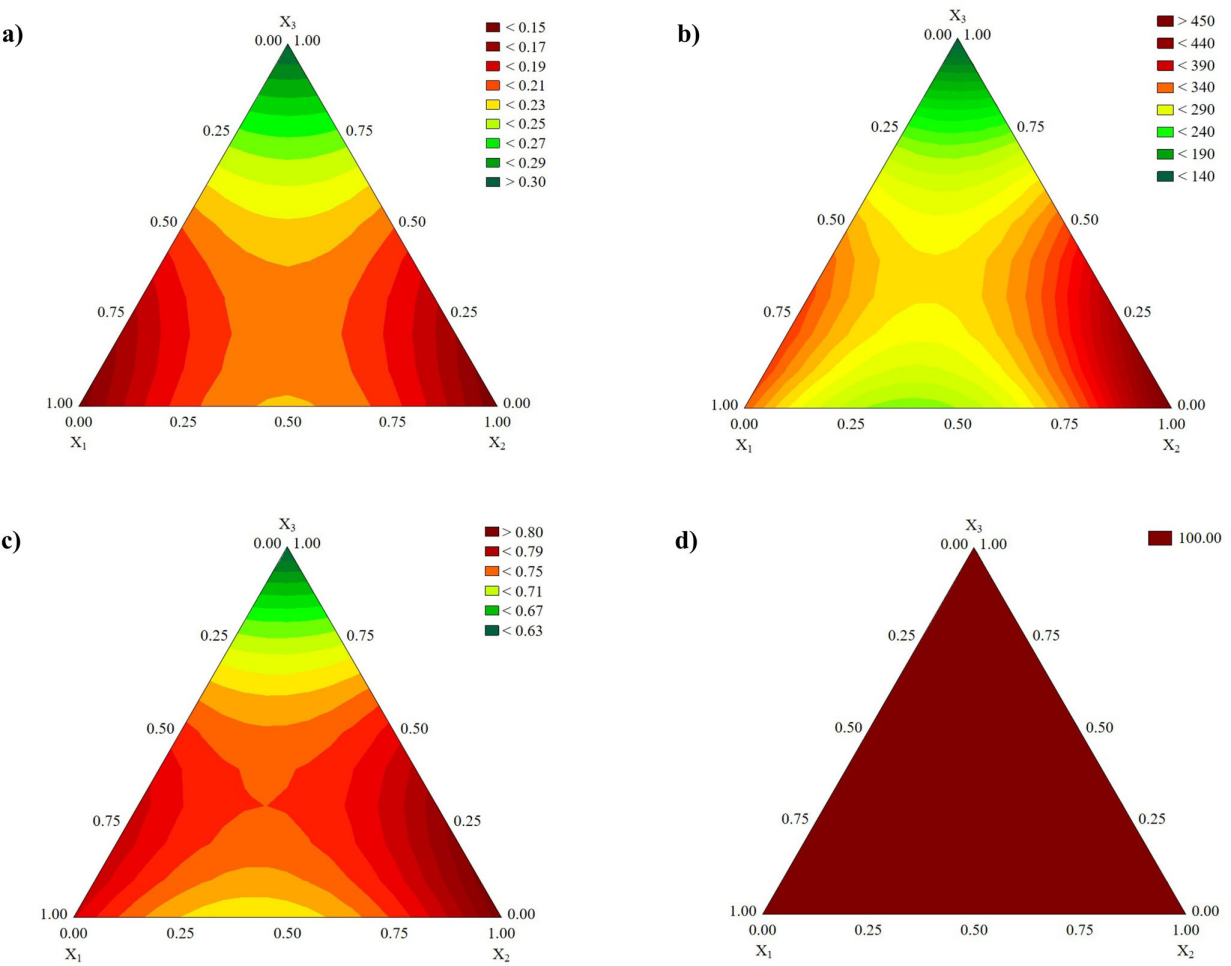
Response surface plots obtained from the simplex‐centroid mixture design for the foam formation properties of pitaya using different protein sources. The evaluated properties were (a) density, (b) overrun, (c) porosity, and (d) stability. The mixture components were *X*_1_ = albumin, *X*_2_ = pea protein and *X*_3_ = soy protein.

**FIGURE 2:**
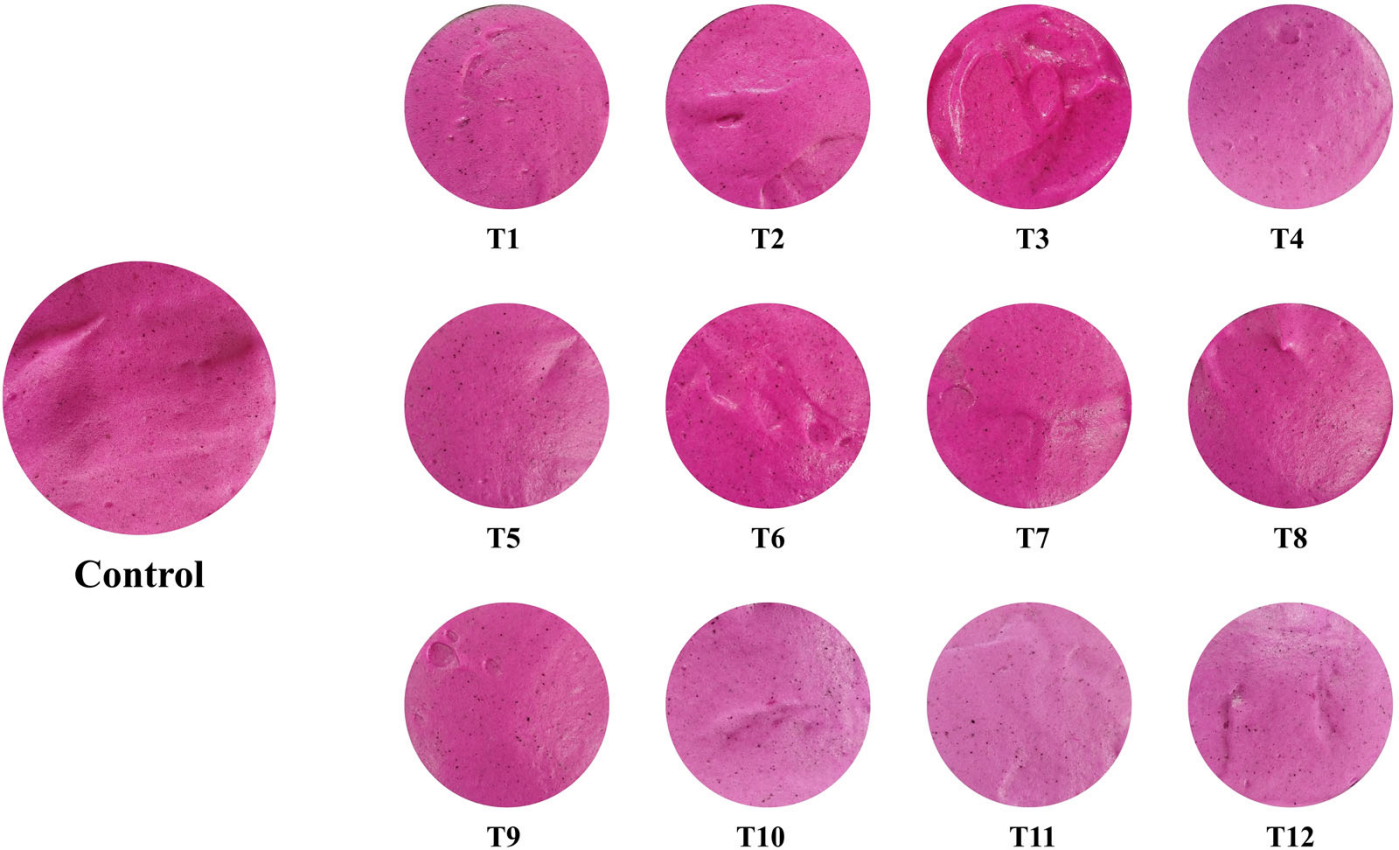
Photographs of all treatments of pitaya foams.

Protein composition had a decisive influence on the physical properties of pitaya foams, as evidenced by the response surfaces obtained from the simplex‐centroid design. The experimental results are presented in Table 1, the response surfaces are shown in Figure 1, the visual appearance of the foam formulations is illustrated in Figure 2, and the fitted quadratic models are summarized in Table 3. The models fitted for density, overrun, and porosity showed statistical significance (*p* < 0.05) and high coefficients of determination (>81%), enabling reliable interpretation of the effects of protein composition on these foam properties. Foam stability was not statistically modeled because no experimental variability was observed among the responses of all evaluated formulations.

**TABLE 3:** Coded equations predicted by the quadratic models of foam parameters and the predicted and experimental values from the optimization.

Foam parameters	Models	*p*‐Value	*R* ^2^ (%)	Predicted values	Experimental values	Control foam
Density	*y* = 0.14*X*_1_ + 0.14*X*_2_ + 0.30*X*_3_ + 0.28*X*_1_ *X*_2_	0.01	86.26	0.14	0.16 ± 0.02 a	0.12 ± 0.01 a
Overrun	*y* = 337.68*X*_1_ + 458.41*X*_2_ + 135.16*X*_3_ − 553.15*X*_1_ *X*_2_	0.03	81.98	458.41	430.75 ± 27.56 a	484.66 ± 25.69 a
Porosity	*y* = 0.77*X*_1_ + 0.82*X*_2_ + 0.63*X*_3_ − 0.28*X*_1_ *X*_2_	0.02	82.91	0.82	0.81 ± 0.01 a	0.82 ± 0.01 a
Stability	n.a	n.a	n.a	n.a	100.00 ± 0.00 a	100.00 ± 0.00 a

*Note: X*_1_, *X*_2_, and *X*_3_ are the coded values. *X*_1_ = albumin, *X*_2_ = pea protein and *X*_3_ = soy protein. Control foam = formulated using exclusively whey protein as a foaming agent. The experimental values are expressed as the mean of triplicates ± standard deviation (*n* = 3). Different letters in the same row indicate a significant difference between treatments (*p* < 0.05). n.a. = not applicable. Foam stability was not statistically modeled because all formulations showed 100.00 ± 0.00% stability, with no liquid drainage during the assay. Therefore, the model, *p*‐value, and *R* ^2^ were not applicable for this response.

Among the evaluated variables, density (Figure 1a) was strongly influenced by the presence of soy protein, with a positive contribution to the model indicating an increase in this parameter as its proportion increased. This behavior may be associated with physicochemical characteristics of soy protein reported in the literature, such as lower solubility, higher hydrophobicity, and aggregation tendency, which can hinder molecular diffusion to the air–liquid interface and may compromise the formation of fine bubbles during whipping (Farid et al. 2023). In contrast, albumin and pea protein showed more favorable effects, with lower contributions to density increase and better air incorporation performance. This response may be related to higher interfacial adsorption capacity and the possible formation of more uniform protein films, as reported for proteins with favorable foaming properties (Brar et al. 2020). Coefficient analysis also indicated that binary mixtures tended to increase density, suggesting a reduction in product lightness compared with the use of pea protein alone.

Overrun (Figure 1b) followed a similar trend, reinforcing that air incorporation efficiency may be associated with the interfacial properties of each protein. The response surface indicated more pronounced effects for albumin and pea protein, whereas soy gradually reduced expansion within the experimental domain. This behavior could be explained, at least in part, by the ability of globular or partially flexible proteins to reduce surface tension and contribute to the initial stabilization of formed bubbles, while more rigid and compact proteins may limit the formation and growth of the aerated system (Çalışkan Koç et al. 2022). The negative coefficient of the *X_1_X_2_* interaction suggests that the combination of albumin and pea protein did not enhance foam expansion beyond their individual effects. This response may indicate possible interfacial competition or interactions in the aqueous phase, which could have reduced bubble formation efficiency and resulted in expansion lower than expected from the sum of the individual effects (Vimercati et al. 2022).

Porosity (Figure 1c) followed the same general trend as density and expansion, reflecting the ability of proteins to support bubble structure and modulate the internal architecture of the foam. The model indicated positive effects of the three proteins individually but a negative interaction between pea and soy proteins, suggesting that combining these plant proteins may have contributed to a less cohesive interfacial matrix or to a less uniform pore distribution, as also discussed by Vimercati et al. (2022) for interactions among other protein complexes. Formulations with lower soy content and moderate proportions of albumin or pea protein tended to produce more porous foams, which may be associated with more open and homogeneous internal structures. These results suggest that the foam formulated with pea protein was highly aerated and light, a desirable characteristic for final product texture, whereas higher soy protein proportions were associated with more compact and less porous foam structures (Brar et al. 2020).

Regarding stability (Figure 1d), an atypical and highly favorable behavior was observed: all experimental formulations, regardless of protein mixture, maintained 100% stability during the evaluated period. This result suggests that, under the tested conditions, foam stability was not primarily governed by the protein mixture but was likely influenced by the pitaya pulp matrix and by the fixed concentration of xanthan gum used in all formulations. Natural fibers and pectins present in the fruit may have increased the viscosity of the continuous phase, reducing liquid drainage and bubble collapse and helping maintain foam structural integrity across all treatments (Jarpa‐Parra et al. 2016). In addition, the use of xanthan gum at the same level in all formulations played a crucial role as a stabilizing agent, contributing to foam structure maintenance and justifying its relevance in different industrial applications (Boddapati et al. 2025). Since all formulations reached the maximum measured stability value, this response did not allow clear discrimination among protein mixtures.

**FIGURE 3:**
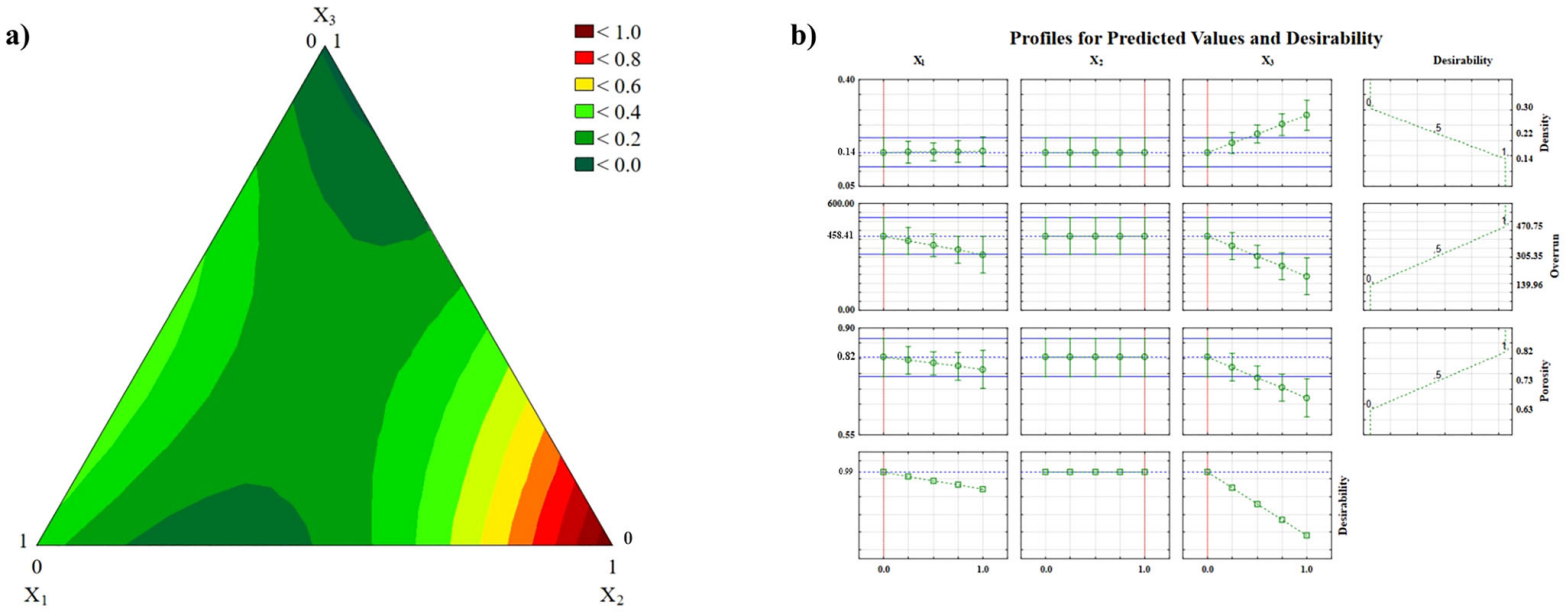
Optimization of the pitaya foam formulation using the desirability function. (a) Overall desirability surface based on the simultaneous minimization of density and maximization of overrun and porosity. (b) Predicted response and desirability profiles for albumin (*X*_1_), pea protein (*X*_2_), and soy protein (*X*_3_). The selected optimum corresponded to *X*_1_ = 0, *X*_2_ = 1, and *X*_3_ = 0.

Based on density, overrun, and porosity (Figure 3a,b), optimization using the desirability method indicated the optimum formulation corresponding to the exclusive use of pea protein (*X*_1_ = 0; *X*_2_ = 1; *X*_3_ = 0). This formulation provided the best balance between air incorporation and foam structure, contributing to low density, high expansion, and high porosity. Foam stability was not included in the desirability function because all formulations remained stable, with no liquid drainage, and therefore this response did not provide discriminatory information for optimization. The absence of soy in the optimum formulation confirms its negative role in responses associated with foam structuring, whereas the absence of albumin reflects the need to balance foaming and stabilizing properties simultaneously. Validation of the optimal formulation showed close agreement between the predicted and experimental values. The chi‐square goodness‐of‐fit test indicated no statistically significant differences, with calculated *χ* ^2^ values of 0.0027 for density, 1.6694 for overrun, and 0.0001 for porosity, all lower than the critical *χ* ^2^ value for 1 degree of freedom at the 95% confidence level (3.8410). Therefore, the fitted models were considered adequate and reliable for predicting the optimal operating condition within the evaluated optimization region (Table 3).

Density, porosity, expansion, and stability values for the optimum formulation (T2) did not differ significantly (*p* > 0.05) from those of the control formulation. Therefore, within the experimental conditions evaluated, no statistically significant differences were detected between the optimized pea protein foam and the whey protein control for the measured foam properties. These results indicate that pea protein showed comparable technological performance to whey protein in forming a structurally stable and homogeneous pitaya foam suitable for subsequent drying. Thus, pea protein may be considered a promising plant‐based alternative foaming agent for pitaya foam‐mat drying, although this finding should be interpreted within the specific formulation and processing conditions used in this study.

### 3.2 Drying Process

#### 3.2.1 Effect of Drying Method

**FIGURE 4:**
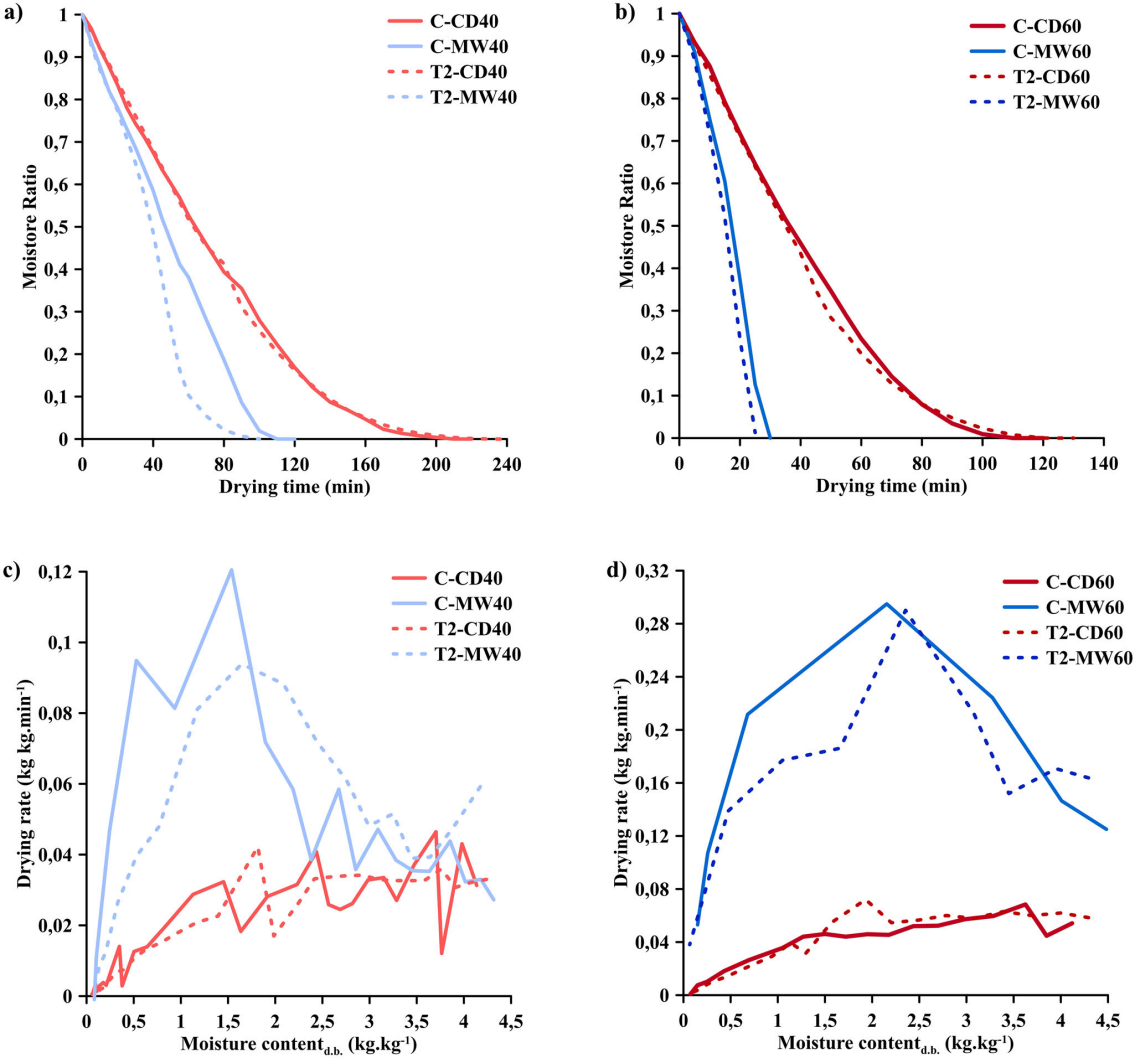
Moisture ratio (a, b) and drying rate (c, d) behavior of pitaya foams dried by convective and microwave methods at 40°C and 60°C. Treatment codes: C = control foam (whey protein); T2 = optimized foam (pea protein); CD = convective drying; MW = microwave drying; 40 and 60 = drying air temperatures (°C).

The drying method significantly affected the drying performance of pitaya foams, directly influencing MR behavior (Figure 4a,b), DR (Figure 4c,d), drying time, apparent effective diffusivity, and energy‐related parameters. The factorial ANOVA showed that drying time was significantly affected by drying method, temperature, and the method × temperature interaction, whereas foam formulation and the interactions involving formulation were not significant (Supplementary Material Table S4). Overall, intermittent microwave drying (MW) was faster than intermittent CD, resulting in higher DRs and shorter processing times (Table 4). For whey‐protein foams, MW reduced drying time by 60.35% at 40°C and 75.00% at 60°C, while reductions of 65.67% and 78.74% were observed for pea‐protein foams at the same temperatures, respectively.

**TABLE 4:** Drying time, energy indicators, apparent effective moisture diffusivity, and statistical indicators of model fitting for pitaya foam‐mat drying.

	C‐CD40	C‐CD60	T2‐CD40	T2‐CD60	C‐MW40	C‐MW60	T2‐MW40	T2‐MW60
**Drying time (min)**	227 ± 21 a	120 ± 10 bc	233 ± 21 a	127 ± 6 b	90 ± 5 cd	30 ± 5 e	80 ± 5 d	27 ± 3 e
**Duty cycle (%)**	29.02 ± 0.89 c	60.72 ± 2.97 a	28.20 ± 1.23 c	57.39 ± 1.12 a	15.13 ± 0.46 d	39.20 ± 0.69 b	14.87 ± 0.46 d	37.73 ± 0.58 b
**Energy consumption (kWh)**	1.51 ± 0.15 a	1.60 ± 0.14 a	1.55 ± 0.15 a	1.69 ± 0.09 a	0.17 ± 0.01 b	0.15 ± 0.03 b	0.15 ± 0.00 b	0.13 ± 0.02 b
**SMER (kg/kWh)**	0.0320 ± 0.0032 b	0.0304 ± 0.0029 b	0.0310 ± 0.0031 b	0.0286 ± 0.0012 b	0.2804 ± 0.0219 a	0.3394 ± 0.0799 a	0.3119 ± 0.0069 a	0.3764 ± 0.0480 a
**SEC (kWh/kg)**	31.4380 ± 3.2379 a	33.1426 ± 3.1331 a	32.4471 ± 3.1258 a	34.9421 ± 1.3969 a	3.5805 ± 0.2744 b	3.0568 ± 0.7068 b	3.2074 ± 0.0711 b	2.6859 ± 0.3402 b
**Apparent D*_eff_* × 10^8^(m^2^/s)**	2.47 ± 0.15c	4.29 ± 0.12 b	2.64 ± 0.11 c	4.28 ± 0.40 b	3.01 ± 0.61 b	10.60 ± 2.16 a	4.51 ± 0.38 b	11.00 ± 2.62 a
***R* ^2^**	0.9448 ± 0.0036 a	0.9429 ± 0.0068 a	0.9517 ± 0.0058 a	0.9454 ± 0.0058 a	0.8522 ± 0.0317 b	0.8687 ± 0.0122 b	0.8757 ± 0.0113 b	0.8787 ± 0.0312 b
**RMSE**	0.1134 ± 0.0048 b	0.1087 ± 0.0054 b	0.1056 ± 0.0077 b	0.1066 ± 0.0067 b	0.1652 ± 0.0152 a	0.1800 ± 0.0070 a	0.1688 ± 0.0102 a	0.1795 ± 0.0104 a
***χ* ^2^**	0.0138 ± 0.0011 c	0.0132 ± 0.0014 c	0.0120 ± 0.0018 c	0.0128 ± 0.0014 c	0.0307 ± 0.0055 b	0.0456 ± 0.0015 a	0.0324 ± 0.0042 b	0.0398 ± 0.0080 ab

*Note*: The values are expressed as the mean of triplicates ± standard deviation (*n* = 3). Different letters in the same row indicate a significant difference between treatments (*p* < 0.05) according to the Tukey test. Variable abbreviations: SMER = specific moisture extraction rate; SEC = specific energy consumption; Apparent *D*_eff_ = apparent effective moisture diffusivity coefficient; *R* ^2^ = determination coefficient; RMSE = root mean square error; *χ* ^2^ = reduced chi‐square. Treatment codes: *C* = control foam (whey protein); T2 = optimized foam (pea protein); CD = convective drying; MW = microwave drying; 40 and 60 = drying air temperatures (°C).

These differences are consistent with the distinct heat and mass transfer mechanisms involved in each process. In CD, moisture removal depends mainly on heat transfer from the drying air to the foam surface and subsequent water migration from the interior to the surface, which generally results in slower drying and may be intensified by surface‐layer resistance during dehydration (Kumar et al. 2019). In contrast, MW promotes internal energy absorption by water molecules, increasing vapor pressure gradients and accelerating moisture transport (De Mendonça et al. 2023; Huang et al. 2021). In foamed systems, this internal vapor generation may affect water‐rich regions, such as liquid lamellae and Plateau borders, contributing to structural expansion, bubble inflation, wall thinning and, under more intense drying conditions, possible microcrack formation or partial foam collapse (De Mendonça et al. 2023; Huang et al. 2021; Gaewsondee et al. 2025).

The drying‐rate curves reflected these differences between methods (Figure 4c,d). CD treatments showed lower and more gradual moisture removal, with an almost constant drying‐rate period during part of the process, suggesting favorable water movement through the porous foam structure before internal resistance increased as moisture content decreased (Dehghannya et al. 2018; Macedo et al. 2021). In contrast, MW samples showed higher DRs and a more pronounced initial increase, especially at 60°C, due to rapid internal heating and enhanced vapor pressure development. After this initial acceleration, the DR decreased as moisture content declined, reducing microwave energy absorption and increasing internal resistance to mass transfer (Turk et al. 2025; Saijuntha et al. 2024). Thus, MW favored faster drying mainly because of its volumetric heating mechanism (De Mendonça et al. 2023; Huang et al. 2021).

The apparent effective moisture diffusivity values supported these observations. Apparent Deff was significantly affected by drying method, temperature, and the method × temperature interaction, while foam formulation was not significant (Supplementary Material Table S4). Higher apparent Deff values were obtained for MW samples than for CD samples under the same temperature conditions (Table 4), indicating greater apparent moisture transport under the adopted Fickian modeling assumptions. This interpretation is consistent with the shorter drying times and higher DRs observed for MW samples (Wang et al. 2019; İzli et al. 2022; Huang et al. 2021). MW also improved energy performance, as indicated by significantly lower SEC, higher SMER, and lower duty cycle compared with CD, suggesting more efficient use of the applied energy under the laboratory‐scale conditions evaluated (Wang et al. 2019; Huang et al. 2021; İzli et al. 2022). Similar relationships among drying configuration, moisture transport, and energy demand have been reported for fruit drying, in which changes in heating intensity, air temperature, or airflow distribution affected drying time, apparent Deff, SEC, thermal efficiency, and physical quality attributes (El‐Mesery et al. 2021, 2023a, 2023b). Therefore, the main advantage of MW in this study was the combined reduction in drying time and energy demand, rather than an isolated effect on moisture diffusivity.

It is important to note that comparisons between the drying methods should be interpreted with caution. In microwave drying, temperature was monitored at a single control point within the foam layer. Thus, the recorded values represent the thermal behavior at the sensor position, rather than the complete spatial temperature distribution within the sample, and the occurrence of local temperature differences cannot be completely ruled out. In addition, the energy results should be interpreted considering the laboratory‐scale configuration used in this study. At larger scales, convective dryers may include improved insulation, air recirculation, and heat recovery systems, which can substantially reduce thermal losses and improve overall energy efficiency.

#### 3.2.2 Effect of Drying Temperature

Drying temperature significantly affected the drying kinetics of pitaya foams, but its effect depended on the drying method. The factorial ANOVA confirmed significant effects of temperature, drying method, and the method × temperature interaction on drying time (Supplementary Material Table S4). Increasing the temperature from 40°C to 60°C accelerated MR reduction (Figure 4a,b), which was directly reflected in shorter drying times for both drying methods (Table 4). This behavior is associated with higher thermal gradients and greater water mobility, which favor apparent moisture transport within the matrix (Qadri 2022; Huang et al. 2021; Macedo et al. 2021; Kalambe and Guhe 2026).

In CD, drying time decreased from approximately 227 to 233 min at 40°C to 120 to 127 min at 60°C, reflecting the effect of higher air temperature on convective heat transfer, surface evaporation, and internal moisture diffusion (Kumar et al. 2019; Lopes et al. 2024; Macedo et al. 2021). In MW, the same temperature increase reduced drying time from 80 to 90 min to only 27 to 30 min, indicating that the combination of higher temperature and microwave energy intensified moisture transport. However, this effect should not be attributed exclusively to volumetric heating, since intermittent operation was used to maintain controlled temperature levels. Under more intense conditions, local temperature heterogeneity, internal vapor pressure development, and structural changes may also increase the risk of overheating and degradation of heat‐sensitive compounds (Kumar et al. 2019).

The temperature effect was also reflected in the drying‐rate curves and Deff values. Higher temperatures increased DRs, particularly during the initial stages of MW drying (Figure 4c,d), and resulted in higher apparent Deff values for both methods (Table 4). In CD, apparent Deff approximately doubled with increasing temperature, which is consistent with processes governed mainly by thermal diffusion under the adopted modeling assumptions (Macedo et al. 2021). In MW, apparent Deff increased more markedly at 60°C, indicating that the combination of higher temperature and microwave application enhanced apparent moisture transport within the material. (Gaewsondee et al. 2025).

From an energy perspective, increasing temperature did not uniformly improve process efficiency. Temperature did not significantly affect total energy consumption or SEC but significantly affected duty cycle and SMER, with a significant method × temperature interaction for SMER (Supplementary Material Table S4). Although higher temperature shortened drying time, the energy required to reach and maintain the target temperature may partially offset this kinetic advantage. This response may be associated with sensible heat losses to the environment and dryer components, meaning that the supplied thermal energy is not fully converted into latent heat of vaporization (Kumar et al. 2019; Qadri 2022). Thus, the improvement in energy efficiency depended on the drying method and operating conditions (Lopes et al. 2024).

This response agrees with previous fruit drying studies showing that higher drying temperatures can accelerate moisture removal and increase apparent Deff, but their effect on energy efficiency depends on the drying configuration and operating conditions (El‐Mesery et al. 2023, El‐Mesery et al. 2023). In addition, hybrid infrared‐CD of apple slices showed that process intensification may reduce drying time and SEC but can also affect quality attributes such as shrinkage, rehydration, and color (El‐Mesery et al. 2021). Overall, while 60°C improved DR and reduced processing time; 40°C may represent a more balanced condition when process efficiency and preservation of thermosensitive compounds are considered together.

#### 3.2.3 Effect of the Foaming Agent

The foaming agent plays an essential role by creating the foam structure needed to accelerate the DR. This occurs because the foam generated by foaming agents has a large surface area and a porous structure, which aids moisture removal from the sample (Akman et al. 2024). Changes in the type of foaming agent may result in distinct foam behaviors due to variations in protein amino acid composition, molecular size, surface polarity, and degree of hydrophobicity (Mulyadi et al. 2024). Consequently, their structural differences can influence DRs and foam‐forming characteristics. For whey, which has a smaller globular structure, there may be greater water‐holding capacity and higher foam expansion, whereas pea protein, with a more complex and larger molecular structure, may present slightly different foam‐forming characteristics (De Paula et al. 2020; Harasym et al. 2025; Phuong et al. 2024).

In drying pitaya foams, the type of foaming agent, whey protein or pea protein, did not significantly affect drying time, energy consumption, SEC, SMER, or apparent effective diffusivity, as confirmed by the factorial ANOVA (Table 4; Supplementary Material Table S4). However, foam formulation significantly affected the duty cycle, indicating that the relative time during which the heating source remained on was influenced by the protein matrix, even though this effect was not strong enough to significantly change total drying time or the main energy‐efficiency indicators. This behavior is consistent with the findings of Akman et al. (2024), who compared egg albumin and whey protein isolate and observed that both supported efficient moisture removal, which agrees with the similar drying behavior observed between whey protein and pea protein foams in the present study.

This similar drying behavior can be explained by interactions between the proteins and the stabilizer used, which play a crucial role in foam formation and stability. Phuong et al. (2024) demonstrated that protein‐stabilizer interactions, such as with maltodextrin, directly influence foam physical properties. Proteins form a viscoelastic layer at the air–water interface, while the stabilizer creates a network that stabilizes this structure. These interactions increase foam surface area, favoring efficient drying and accelerating moisture removal. In the present study, the whey protein control and optimized pea‐protein foam did not differ significantly in the main foam properties evaluated (Table 3). Therefore, xanthan gum may have contributed to similar drying behavior by reducing the effect of protein‐specific structural differences (Boddapati et al. 2025). This pattern is consistent with the findings of Phuong et al. (2024), who observed similar drying behaviors between soy protein and egg albumin due to the stabilizing action of maltodextrin.

The results of this study indicate that foam drying behavior was predominantly controlled by drying method and temperature, as also indicated by the significant effects and interactions observed for drying time, apparent Deff, SMER, and duty cycle. Foam formulation had a more limited effect on the drying stage, mainly affecting duty cycle, but not drying time, energy consumption, SEC, SMER, or apparent Deff. Therefore, under the specific experimental conditions evaluated, particularly the use of 5% (w/w) foaming protein and 1% (w/w) xanthan gum in pitaya foam formulations, whey protein and pea protein produced foams with comparable drying performance. These findings support the potential use of pea protein as an alternative foaming agent for pitaya foam‐mat drying, without implying that pea protein will necessarily perform equivalently to whey protein in all foam‐mat drying systems.

### 3.3 Microstructural Analysis

**FIGURE 5:**
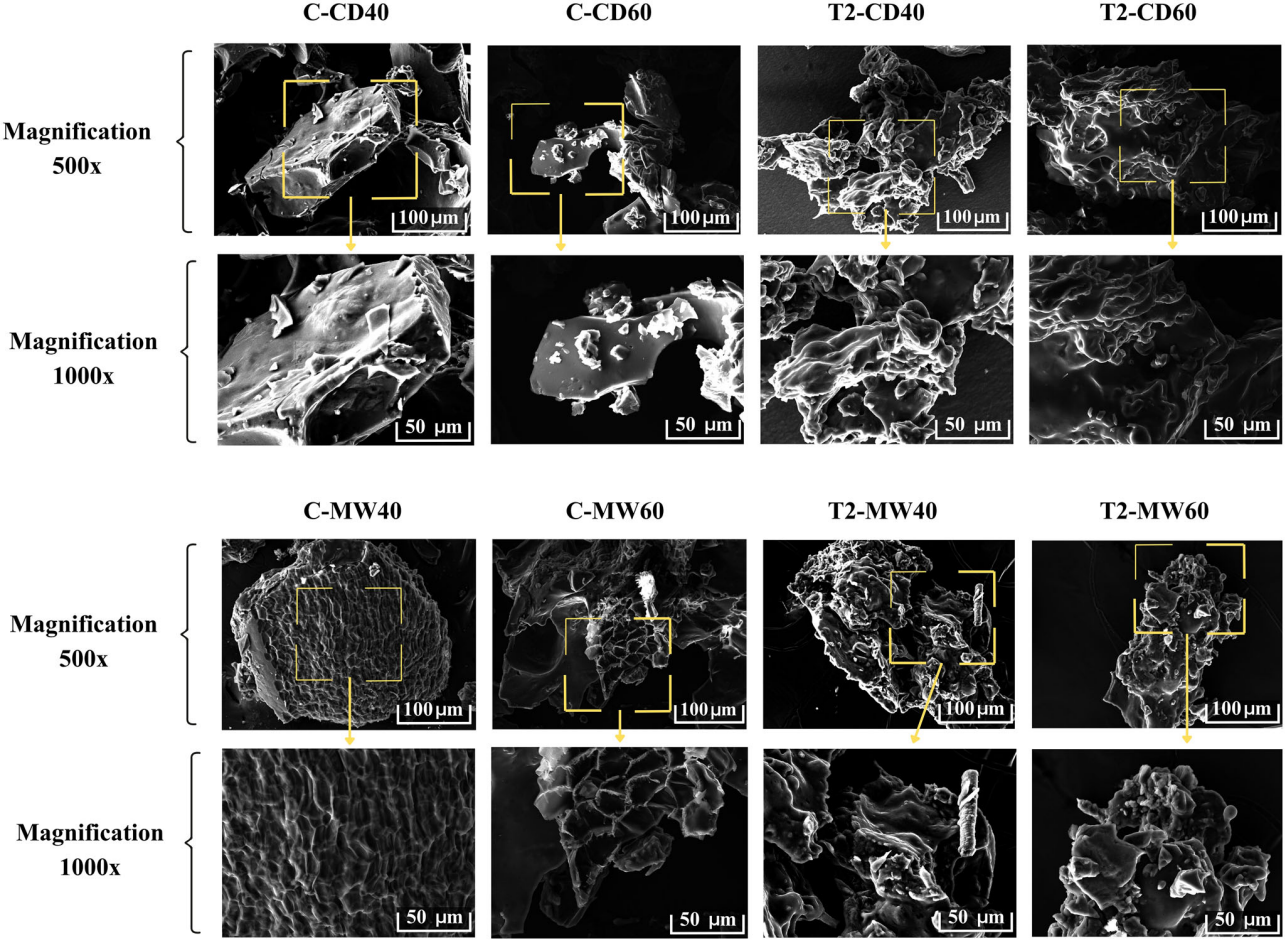
Scanning electron microscopy micrographs of pitaya powders. Scale bars correspond to 100 µm at 500× magnification and 50 µm at 1000× magnification. Yellow highlights indicate the magnified regions. Treatment codes: C = control foam (whey protein); T2 = optimized foam (pea protein); CD = convective drying; MW = microwave drying; 40 and 60 = drying air temperatures (°C).

SEM (Figure 5) evidenced marked differences in pitaya powder microstructure, highlighting the influence of dryer type, drying temperature, and foam formulation on particle morphology. In general, micrographs revealed irregularly shaped particles with surfaces ranging from relatively smooth and compact to highly rough and porous, reflecting distinct moisture‐removal mechanisms and varying degrees of structural collapse associated with processing conditions.

Powders obtained by CD consistently exhibited more compact, collapsed structures, characterized by relatively smooth surfaces, fewer open pores, and densified regions, especially in samples dried at 60°C. This behavior is typical of processes dominated by surface heat transfer, in which gradual water evaporation favors progressive collapse of the foamed matrix over drying time, resulting in denser and less porous particles (Lopes et al. 2024). In contrast, microwave‐assisted samples exhibited significantly more porous, irregular, and fragmented microstructures, with rough surfaces and internal cavities. Volumetric microwave heating promotes rapid water vaporization within the matrix, generating pressure gradients that favor expansion and rupture of the foamed structure before complete collapse occurs, resulting in particles with lower compaction and higher porosity (Santos et al. 2025a).

The influence of drying temperature was clearly observed within each technology. At 40°C, both convective and microwave‐assisted drying resulted in particles with relatively well‐preserved structures, with more defined walls and a lower degree of collapse, indicating milder drying. In contrast, increasing the temperature to 60°C intensified structural collapse and coalescence, resulting in more densely packed surfaces in CD and more fragmented, irregular structures in microwave drying. These results indicate that, although higher temperatures accelerate drying kinetics, they increase thermal severity and negatively affect powder microstructural integrity (Silveira et al. 2024).

Regarding foam formulation, micrograph analysis revealed clear morphological differences between powders formulated with whey protein (control) and pea protein (T2), even under the same drying conditions. Control samples tended to exhibit more compact, continuous structures, with laminar regions and more homogeneous surfaces, indicating greater cohesion of the protein matrix after moisture removal. Conversely, powders formulated with pea protein displayed rougher, more irregular, and fragmented surfaces, with greater structural heterogeneity and the presence of discontinuities and cavities, suggesting a less uniform collapse of the foamed matrix during drying. These results demonstrate that the nature of the protein used as a foaming agent influences the organization of the dried matrix at the microscopic scale, modulating the final particle morphology and potentially impacting rehydration, density, and moisture interactions.

### 3.4 Physicochemical Parameters

The yield of the dried pitaya samples was not significantly affected by drying method, temperature, foam formulation, or their interactions (Table 5; Supplementary Material Table S5). This behavior indicates that total solids recovery remained similar among treatments, regardless of the processing condition applied. Considering that the raw material was standardized, the amount of foaming agent did not vary, and physical losses during processing were minimized, the final yield depended mainly on the initial solids content of the fruit and incorporated additives. This confirms that all drying conditions were efficient in recovering the dried product, with yields close to 50% (Table 5), comparable to results obtained using more advanced and high‐cost technologies such as spray drying (Costa et al. 2025).

**TABLE 5:** Physicochemical characteristics of pitaya powders.

	C‐CD40	C‐CD60	T2‐CD40	T2‐CD60	C‐MW40	C‐MW60	T2‐MW40	T2‐MW60
**Yield (%)**	49.69 ± 0.60 a	49.70 ± 0.81 a	50.12 ± 0.36 a	49.86 ± 0.98 a	49.64 ± 0.28 a	48.87 ± 0.69 a	50.24 ± 1.03 a	49.10 ± 0.23 a
$\rho_{B}$ **(g/cm^3^)**	0.305 ± 0.018 bc	0.323 ± 0.024 b	0.396 ± 0.006 a	0.335 ± 0.008 b	0.246 ± 0.007 d	0.283 ± 0.006 c	0.229 ± 0.004 d	0.235 ± 0.0022 d
$\rho_{T}$ **(g/cm^3^)**	0.355 ± 0.021 b	0.384 ± 0.010 b	0.442 ± 0.015 a	0.386 ± 0.007 b	0.276 ± 0.008 cd	0.299 ± 0.007 c	0.261 ± 0.006 d	0.261 ± 0.0060 d
**CI (%)**	13.99 ± 3.25 a	15.75 ± 4.19 a	10.41 ± 2.37 ab	13.15 ± 3.07 a	10.57 ± 1.42 ab	5.56 ± 0.15 b	12.13 ± 1.33 ab	10.01 ± 1.41 ab
**HR**	1.16 ± 0.04 a	1.19 ± 0.06 a	1.12 ± 0.03 ab	1.15 ± 0.04 ab	1.12 ± 0.02 ab	1.06 ± 0.00 b	1.14 ± 0.02 ab	1.11 ± 0.02 ab
**WSI (%)**	43.75 ± 2.25 ab	49.68 ± 1.17 a	38.57 ± 2.72 bc	39.06 ± 2.35 bc	40.21 ± 3.15 bc	35.80 ± 2.69 c	35.99 ± 0.93 c	28.48 ± 0.90 d
**WAI (g/g)**	4.19 ± 0.56 cde	3.84 ± 0.11 e	4.06 ± 0.19 de	4.28 ± 0.30 bcde	5.21 ± 0.44 abc	5.95 ± 0.50 a	4.91 ± 0.04 abcd	5.27 ± 0.44 ab
**Hygroscopicity (g/g)**	0.205 ± 0.008 abc	0.237 ± 0.006 a	0.195 ± 0.005 bcd	0.216 ± 0.012 ab	0.141 ± 0.013 e	0.170 ± 0.017 de	0.133 ± 0.021 e	0.166 ± 0.011 cde
**Wettability (s)**	130 ± 10 ab	150 ± 15 a	95 ± 5 cd	82 ± 13 de	138 ± 8 ab	122 ± 10 bc	65 ± 5 e	57 ± 8 e
**Moisture (%)**	7.62 ± 1.61 a	8.29 ± 1.94 a	7.95 ± 0.20 a	7.57 ± 0.79 a	7.42 ± 0.21 a	6.58 ± 2.10 a	8.35 ± 1.21 a	6.92 ± 1.22 a
***a*_w_**	0.493 ± 0.046 a	0.452 ± 0.065 a	0.392 ± 0.027 a	0.396 ± 0.047 a	0.418 ± 0.037 a	0.392 ± 0.032 a	0.434 ± 0.013 a	0.450 ± 0.040 a

*Note*: The values are expressed as the mean of triplicates ± standard deviation (*n* = 3). Different letters in the same row indicate a significant difference between treatments (*p* < 0.05) according to the Tukey test. Variable abbreviations: *ρ_B_* = bulk density; *ρ_T_* = tapped density; CI = carr index; HR = hausner ratio; WSI = water solubility index; WAI = water absorption index; aw = water activity. Treatment codes: C = control foam (whey protein); T2 = optimized foam (pea protein); CD = convective drying; MW = microwave drying; 40 and 60 = drying air temperatures (°C).

Bulk and tapped densities are essential parameters for characterizing dehydrated products, reflecting the volumetric organization of the material and its total porosity. Bulk density is particularly important for food powders because it affects rehydration, packaging, transport, and commercial acceptance and is influenced by particle size distribution, moisture content, and particle morphology. Tapped density, in turn, indicates the ability of the powder bed to reorganize under mechanical tapping, with lower values generally associated with higher porosity and lower packing efficiency (Akman et al. 2024; Araújo et al. 2022). The factorial ANOVA showed that the drying method significantly affected both bulk and tapped densities, whereas temperature and foam formulation were not significant as isolated factors. However, significant interaction effects were observed, indicating that density changes also depended on specific combinations of drying method, temperature, and formulation (Supplementary Material Table S5). Therefore, the drying method was the main factor affecting powder density, but its effect was modulated by the other process variables.

Samples subjected to microwave drying showed the lowest bulk and tapped density values. This behavior is consistent with the SEM micrographs, which revealed more porous, irregular, and fragmented structures, with rough surfaces and internal cavities in microwave‐dried powders. The volumetric nature of microwave heating may promote localized vapor generation and internal pressure development within the foam matrix, favoring expansion and partial rupture of the structure before complete collapse occurs. As a result, the particles occupy a larger volume and present lower packing efficiency, explaining the lower density values observed for these treatments (Omidi et al. 2024; Silveira et al. 2024; Santos et al. 2025a). In contrast, CD resulted in powders with higher densities, which agrees with the more compact and collapsed structures observed by SEM. These samples showed relatively smooth surfaces, fewer open pores, and densified regions, especially at 60°C. This morphology is consistent with the gradual surface‐driven water removal typical of CD, which favors progressive shrinkage and collapse of the foamed matrix, resulting in denser and less porous particles (Lopes et al. 2024; Ozcelik et al. 2024).

Regarding flow properties, the HR and CI are commonly used in industry to minimize potential problems during powder handling and processing. The factorial ANOVA showed that both indices were significantly affected by the drying method and by the method × temperature and method × formulation interactions, indicating that flow‐related parameters were influenced by the way in which drying conditions modified powder structure. Nevertheless, although statistical differences were detected, the values remained within ranges associated with satisfactory or acceptable flow behavior (Table 5; Supplementary Material Table S5). Good flowability is commonly associated with HR and CI values below 1.18% and 15%, respectively, whereas poor flowability corresponds to values above 1.35% and 26%. Values between these thresholds are considered acceptable (Salgado‐Aristizabal et al. 2025). Thus, the microstructural changes observed by SEM affected the density of the powders more clearly than their practical flow behavior, suggesting that differences in porosity, collapse, and particle organization were not sufficient to impair powder flow under the conditions evaluated.

WSI and WAI are essential parameters to assess the ability of food powders to dissolve in water and retain moisture, respectively (Bogusz et al. 2024; Hossain et al. 2024). Hygroscopicity reflects the ability of a powder to absorb moisture from the environment, thereby affecting its stability during storage. Lower hygroscopicity values indicate greater resistance to changes in physicochemical and sensory properties (Costa et al. 2025; Vimercati et al. 2022). In addition, wettability is important for understanding how powders disperse in liquids and how easily they become hydrated upon contact with water (Araújo et al. 2022). The factorial ANOVA showed that these reconstitution‐related properties were affected differently by drying method, temperature, and formulation (Supplementary Material Table S5).

For WSI, significant effects were observed for drying method and foam formulation, as well as for the method × temperature and temperature × formulation interactions. Microwave‐dried samples, particularly those dried at 60°C, showed lower solubility than convectively dried samples under the same formulation conditions. This suggests that the structural changes promoted by microwave drying, especially when combined with higher temperature, may have reduced the fraction of soluble material released into water. In contrast, WAI was significantly affected by the drying method and by the method × formulation interaction, with microwave‐dried powders generally showing higher water absorption. This response may be associated with the formation of porous and irregular structures, which can favor water retention within the particles. The observed increase in water absorption can be explained by the sponge‐like effect, in which water is retained within particles due to capillary forces or swelling of the protein network (Juarez‐Enriquez et al. 2022). However, this effect was not necessarily accompanied by greater solubilization.

Hygroscopicity was significantly affected by drying method and temperature, but not by foam formulation. Microwave‐dried powders showed lower hygroscopicity than convectively dried powders, indicating lower affinity for environmental moisture. This behavior may be related to structural modifications induced during drying, including changes in surface characteristics and possible exposure of less hydrophilic regions, which may reduce interaction with water vapor and favor storage stability by limiting moisture uptake and agglomeration (Nunes et al. 2022). In contrast, increasing the temperature from 40°C to 60°C tended to increase hygroscopicity, indicating that temperature also contributed to changes in water affinity.

Wettability was significantly affected by drying method and foam formulation, as well as by the method × formulation and method × temperature × formulation interactions. These results indicate that wetting behavior depended on the combined effects of powder structure and protein matrix. Powders formulated with pea protein generally showed shorter wetting times, suggesting faster interaction between the particles and water. This may be associated with differences in particle structure, surface characteristics, and the intrinsic properties of the foaming protein, since powder particles must overcome water surface tension to favor interaction between water and the solid phase (Akman et al. 2024; Araújo et al. 2022; Gao et al. 2022). However, this formulation effect was not observed for all water‐related properties, since WAI and hygroscopicity were not significantly affected by formulation as an isolated factor.

Moisture content indicates the total amount of water present in the product, while water activity reflects the water available for microbiological processes and chemical reactions. Reducing these parameters increases shelf life and facilitates transport and storage, thereby reducing waste and costs (Macedo et al. 2020). Final moisture content was not significantly affected by drying method, temperature, formulation, or their interactions, indicating that the drying conditions were effective in reaching comparable final moisture levels. For water activity, no significant main effects were observed, although the method × formulation interaction was significant. Despite this statistical interaction, all water activity values remained below 0.60, which minimizes the risk of microbial proliferation and contributes to product stability (Cruz et al. 2025a). Therefore, under the experimental conditions used, the dried pitaya powders presented favorable characteristics for conservation, provided that appropriate hygienic conditions, packaging, and storage practices are maintained (Vimercati et al. 2022).

Compared with other foam‐mat dried red pitaya and red pitaya‐based powders, the samples obtained in the present study showed similar stability‐related characteristics, since all water activity values remained below the limit generally associated with microbiological stability. However, the WSI values were lower than those reported for some red pitaya powders formulated with higher proportions of foaming agents, maltodextrin, or protein hydrolysates (Araújo et al. 2022; Akman et al. 2024; Rahayu et al. 2024). This difference may be associated with the use of pitaya pulp containing seeds and with the lower proportion of highly soluble carrier materials in the present formulations.

### 3.5 Color Parameters

**FIGURE 6:**
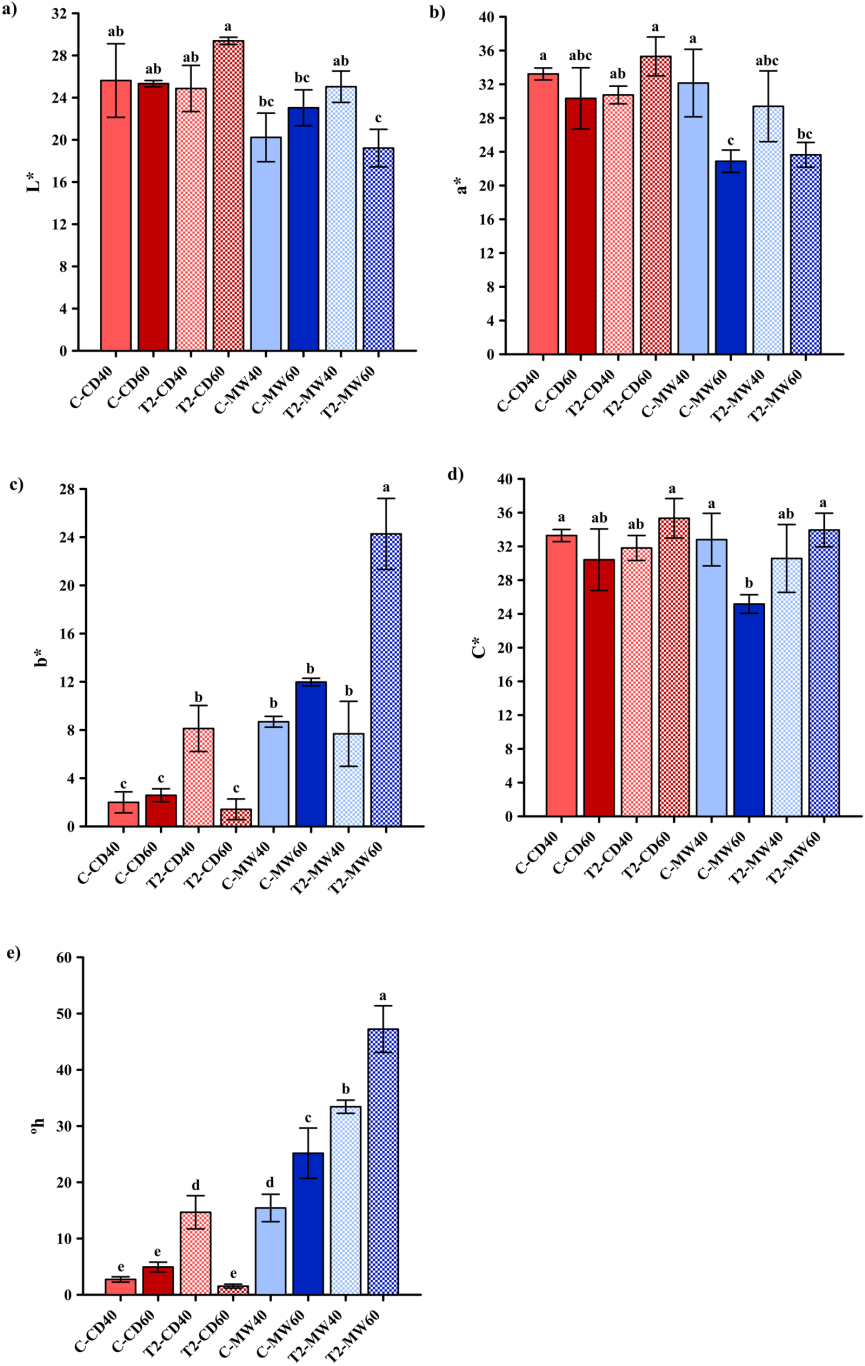
Color parameters of the pitaya powders. The parameters evaluated include (a) lightness, (b) a* (red–green axis), (c) b* (yellow–blue axis), (d) chroma, and (e) hue angle. The bars represent the mean of triplicates ± standard deviation (*n* = 3). Different letters indicate a significant difference between treatments (*p* < 0.05) according to the Tukey test. Treatment codes: *C* = control foam (whey protein); T2 = optimized foam (pea protein); CD = convective drying; MW = microwave drying; 40 and 60 = drying air temperatures (°C).

**FIGURE 7:**
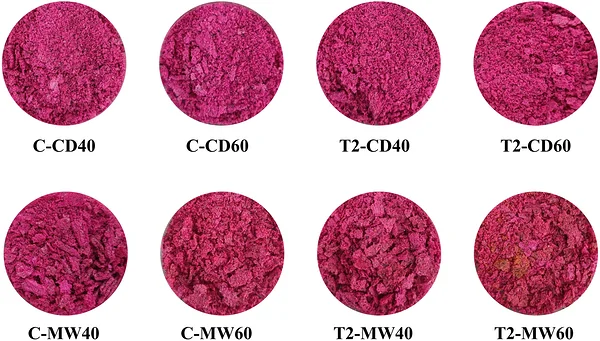
Photographs of the treatments after drying. Treatment codes: C = control foam (whey protein); T2 = optimized foam (pea protein); CD = convective drying; MW = microwave drying; 40 and 60 = drying air temperatures (°C).

Color parameter results for pitaya powders showed that the chromatic properties of the dried products were affected by drying method, temperature, and foam formulation. These effects are shown by the treatment means presented in Figure 6 and Supplementary Material Table S6, visually supported by the powder images in Figure 7 and statistically confirmed by the factorial ANOVA presented in Supplementary Material Table S7. However, the effect of each factor depended on the color coordinate evaluated. For L*, drying method was significant, whereas temperature and formulation were not significant as isolated factors. Nevertheless, the significant method × temperature and method × temperature × formulation interactions indicated that lightness was influenced by specific combinations of drying method, temperature, and formulation. For a*, the drying method and temperature were significant, as were the method × temperature and temperature × formulation interactions, showing that red intensity was mainly affected by the interaction among processing conditions, rather than by formulation alone.

The most evident color changes were observed for b* and *h*°, for which the drying method, temperature, formulation, and all interaction effects were significant. This confirms that the shift toward yellowish tones was governed by the combined effects of process conditions and the protein matrix. Among the treatments, T2‐MW60 showed the most evident chromatic change, with the lowest L* value, one of the lowest a* values, and the highest b* and *h*° values. This indicates that the combination of pea protein, microwave drying, and 60°C produced darker powders, with lower red intensity and a greater shift toward yellowish tones. Thus, the chromatic changes observed in pitaya powders should not be attributed only to the drying method but rather to its interaction with temperature and foam formulation.

This behavior may be associated with the combined effects of drying method, temperature, exposure time, and formulation, since microwave drying at 60°C may modify the real thermal history of the samples and promote localized thermal effects, moisture redistribution, and structural changes that contribute to color changes, including pigment degradation and possible browning reactions (Diógenes et al. 2022). Therefore, although microwave drying reduced processing time, its effect on color was condition‐dependent. At 40°C, the powders generally showed better preservation of the characteristic reddish color of pitaya, whereas the most pronounced color changes occurred at 60°C, especially under microwave drying. This indicates that the visual quality of pitaya powders depends on the balance among DR, thermal exposure, local temperature heterogeneity, and formulation effects (Araújo et al. 2022).

Foam formulation also affected the chromatic characteristics of the powders, but its influence was not uniform across all color parameters. Formulation was significant for b*, C*, and *h*°, while significant interactions involving formulation were also observed, especially for b* and *h*°. The T2 formulation, prepared with pea protein, contributed to distinct color responses, particularly under microwave drying at 60°C, where the highest b* and *h*° values indicated a more intense shift toward yellowish tones. These differences may be related to the intrinsic color of the protein sources and their interaction with the drying method and temperature. Whey protein, with a whitish‐yellow tone, and pea protein, with a greenish‐yellow color, may influence the final color of the powders even at low concentrations, particularly under more severe drying conditions (Akman et al. 2024).

Compared with other red pitaya powders produced by foam‐mat drying, the powders obtained in this study showed lower L* values, indicating darker and more intense red–purple coloration (Akman et al. 2024; Rahayu et al. 2024). This difference may be related to the higher relative contribution of pitaya solids and betalain‐rich pulp, as well as to the lower dilution effect from carrier materials. Previous studies reported that increasing the proportion of foaming agents or carriers can increase powder lightness and modify a* and b* values due to pigment dilution and to the intrinsic color of the added materials (Akman et al. 2024; Mulyadi et al. 2024).

**FIGURE 8:**
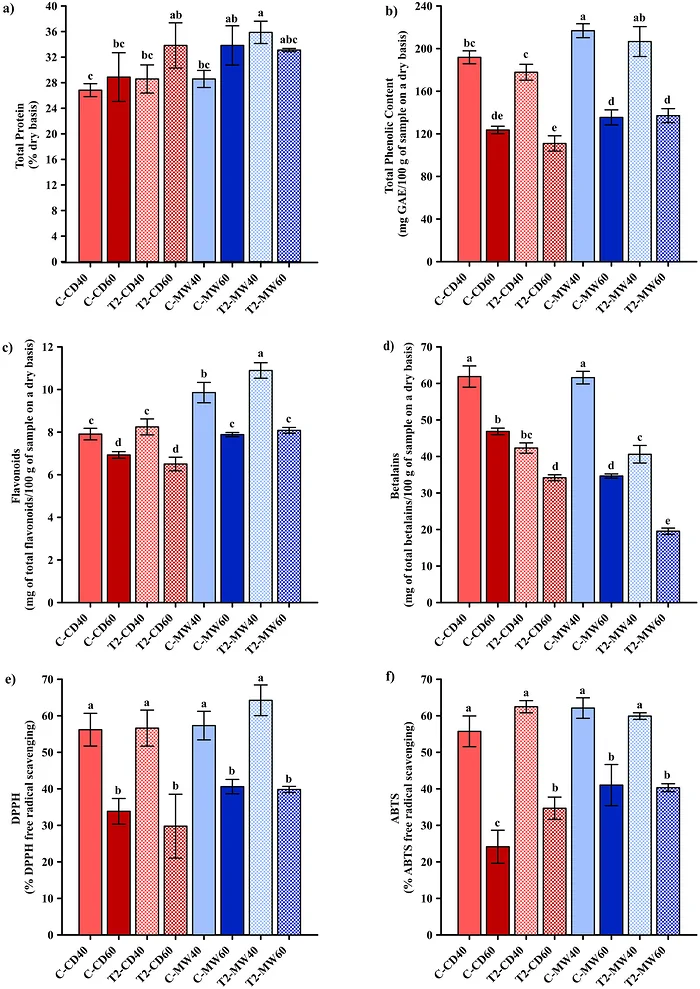
Protein content and bioactive compounds of pitaya powders obtained under different drying treatments. The evaluated parameters include (a) total proteins, (b) total phenolics determined by the Fast Blue method, (c) total flavonoids, (d) betalain content, (e) antioxidant activity by DPPH, and (f) antioxidant activity by ABTS. The bars represent the mean ± standard deviation (*n* = 3). Different letters indicate a significant difference between treatments (*p* < 0.05) according to the Tukey test. Treatment codes: C = control foam (whey protein); T2 = optimized foam (pea protein); CD = convective drying; MW = microwave drying; 40 and 60 = drying air temperatures (°C).

The color changes can also be understood in relation to the betalain results (Figure 8), since these pigments are largely responsible for the characteristic red–purple color of pitaya powders (Figure 7). In treatments with lower red intensity and higher b* and *h*° values, especially at 60°C, the color shift may be related to partial betalain degradation or to changes in the relative contribution of betacyanins and betaxanthins. While betacyanins are mainly associated with red–purple tones, betaxanthins are more closely related to yellow–orange hues. In addition, possible copigmentation effects may have contributed to the final color expression of the powders, since betalains can interact with phenolic compounds and other matrix components, potentially affecting pigment stability and chromatic expression (Martinez et al. 2024; Shah et al. 2023). Drying conditions may affect these pigment–copigment interactions by modifying pigment stability, matrix organization, and the availability of potential copigments.

Therefore, the decrease in a*, together with the increase in b* and *h*° observed in some treatments, suggests that drying may have affected pigment stability and, consequently, the final color expression of the powders. Overall, the chromatic behavior of pitaya powders was governed by the combined effects of drying method, temperature, foam formulation, betalain retention, and possible copigmentation effects. Since copigmentation was not directly measured in this study, it should be considered as a complementary explanation for the observed color differences among treatments.

### 3.6 Protein Content and Bioactive Compounds

The factorial ANOVA showed that drying method and temperature significantly affected protein content, phenolics, flavonoids, betalains, and antioxidant activity determined by DPPH and ABTS assays (Figure 8; Supplementary Material Tables S8 and S9). Foam formulation also influenced most responses, except DPPH. These results indicate that the quality of pitaya powders was not determined by a single processing factor but by the combined effects of drying method, drying temperature, and protein formulation. Overall, temperature was one of the most decisive factors, since treatments performed at 40°C generally showed higher contents of phenolics, flavonoids, and betalains, as well as higher antioxidant activity than those carried out at 60°C. This trend was particularly evident for phenolics, flavonoids, betalains, DPPH, and ABTS, confirming the sensitivity of these compounds and antioxidant responses to increasing drying temperature.

For phenolic compounds, only the main effects of drying method, temperature, and formulation were significant, with no significant interaction effects. This indicates that each factor independently affected phenolic content. Microwave‐dried powders showed higher phenolic contents than convectively dried powders under the same temperature and formulation conditions, which may be associated with the shorter drying time and reduced overall exposure of the matrix to thermal conditions. Although the shorter processing time may have limited the degradation of these compounds, drying‐induced structural changes may also have influenced solvent–matrix interactions and, consequently, the measured contents. Similarly, flavonoid content was higher in microwave‐dried samples, especially at 40°C, with the highest value observed for T2‐MW40. However, unlike phenolics, flavonoids showed significant method × temperature, method × formulation, and temperature × formulation interactions, whereas the three‐way interaction was not significant. These results indicate that flavonoid content depended more strongly on the combination of processing conditions and the protein matrix (Narra et al. 2024).

Betalain content showed a distinct and condition‐dependent response. Drying method, temperature, and formulation exerted significant effects, as did the method × temperature and temperature × formulation interactions. In contrast, the method × formulation and three‐way interactions were not significant. At 40°C, convective and microwave drying resulted in similar betalain contents within each formulation. However, at 60°C, microwave‐dried samples showed lower betalain contents than the corresponding convectively dried samples, with a more pronounced reduction in the T2 formulation. The lowest content was observed for T2‐MW60. These results indicate that the unfavorable effect of microwave drying on betalain content became more evident at 60°C and that the response to increasing temperature was modulated by foam formulation.

This behavior is consistent with previous studies on red pitaya and pigment‐rich powders obtained by foam‐mat drying, in which betalain content and color stability were strongly affected by drying temperature, exposure time, and formulation composition. Araújo et al. (2022) reported that lower drying temperature favored betanin and betaxanthin contents in red pitaya powders, while Firdhauzi et al. (2025) highlighted the importance of foam‐mat drying as a strategy to stabilize betacyanin‐rich dragon fruit peel extracts. Similarly, studies with red dragon fruit powdered drinks showed that foaming‐agent type and foam stability influenced phenolic compounds, flavonoids, and antioxidant activity (Mulyadi et al. 2024; Rahayu et al. 2024). Thus, the higher contents of phenolics, flavonoids, and betalains, as well as the higher antioxidant activity generally observed at 40°C in the present study, agree with the general trend reported for pigment‐rich foam‐mat dried powders. In contrast, the lower betalain contents obtained at 60°C confirm the greater susceptibility of these pigments under more severe processing conditions.

Therefore, the effects of microwave drying should be interpreted with caution and should not be attributed exclusively to volumetric heating. Since the process was conducted intermittently to maintain controlled temperature levels, the observed responses may reflect the balance between shorter drying time, possible local temperature heterogeneity, non‐uniform heating, internal vapor pressure development, and structural changes in the dried matrix. Under microwave conditions, internal vapor pressure may promote expansion, rupture of the foam structure, and the formation of more porous particles (De Mendonça et al. 2023; Huang et al. 2021; Santos et al. 2025a). These structural modifications may alter solvent–matrix interactions and contribute to differences in the measured contents of phenolics and flavonoids. This effect may occur simultaneously with a possible reduction in degradation resulting from the shorter drying time.

For betalains, both thermal degradation and oxidative reactions may have contributed to the lower contents observed at 60°C. Increasing temperature may accelerate reactions such as aldimine bond hydrolysis, isomerization, decarboxylation, and dehydrogenation, resulting in loss of color intensity and the possible formation of yellowish or brownish compounds (Sadowska‐Bartosz and Bartosz 2021). Thermal degradation of betalains is frequently described by first‐order kinetic models, although its rate depends on the matrix, temperature range, exposure time, pH, water activity, and presence of oxygen (Wijesekara and Xu 2024). In addition, oxygen exposure during processing may intensify oxidative reactions and accelerate pigment loss. In powders and encapsulated systems, this susceptibility may also be influenced by particle porosity, residual moisture, and matrix integrity, which affect oxygen diffusion and availability within the material (Yeasmen et al. 2026).

The antioxidant assays also reflected the combined effects of temperature, drying method, and formulation. DPPH was significantly affected only by drying method and temperature, with no significant effect of formulation or interactions. This suggests that DPPH antioxidant activity was mainly governed by thermal intensity and the drying process, with higher values generally observed at 40°C. In contrast, ABTS was significantly affected by drying method, temperature, and formulation, as well as by the method × temperature and method × formulation interactions, indicating greater sensitivity to the combined effects of processing conditions and the protein matrix (Gao et al. 2022). These differences may be related to the chemical nature and accessibility of antioxidant compounds after drying, since DPPH and ABTS radicals may respond differently to phenolics, flavonoids, betalains, and other antioxidant molecules present in pitaya powders (Mendonça et al. 2022; Gulcin 2025; Knez et al. 2025).

Protein content was also affected by drying method, temperature, and formulation, and the significant three‐way interaction indicated that the effect of formulation depended simultaneously on drying method and temperature. The highest protein values were observed mainly in microwave‐dried powders formulated with pea protein, particularly at 40°C. This response may be associated with the contribution of the protein source to the dried matrix and with changes in powder structure during drying. Overall, the results show that bioactive compound contents, antioxidant activity, and protein content in pitaya powders were influenced by drying conditions and foam formulation, rather than by the isolated effect of microwave heating, temperature, or foaming‐agent type (Araújo et al. 2022; Costa et al. 2025).

When drying performance and bioactive responses are analyzed together, microwave drying showed a condition‐dependent effect. The shorter drying time was associated with lower SEC, higher SMER, higher phenolic and flavonoid contents, and greater antioxidant activity, especially at 40°C. Under these milder conditions, the shorter processing time may have limited the overall exposure of the samples to thermal conditions. However, at 60°C, the lower betalain contents, particularly in T2‐MW60, indicate that the kinetic and energy advantages of microwave drying were not necessarily accompanied by the maintenance of all thermosensitive compounds. Therefore, microwave drying improved process efficiency, but its effect on bioactive compounds depended on the balance among drying time, temperature, possible local thermal heterogeneity, and foam formulation.

### 3.7 Principal Component Analysis

**FIGURE 9:**
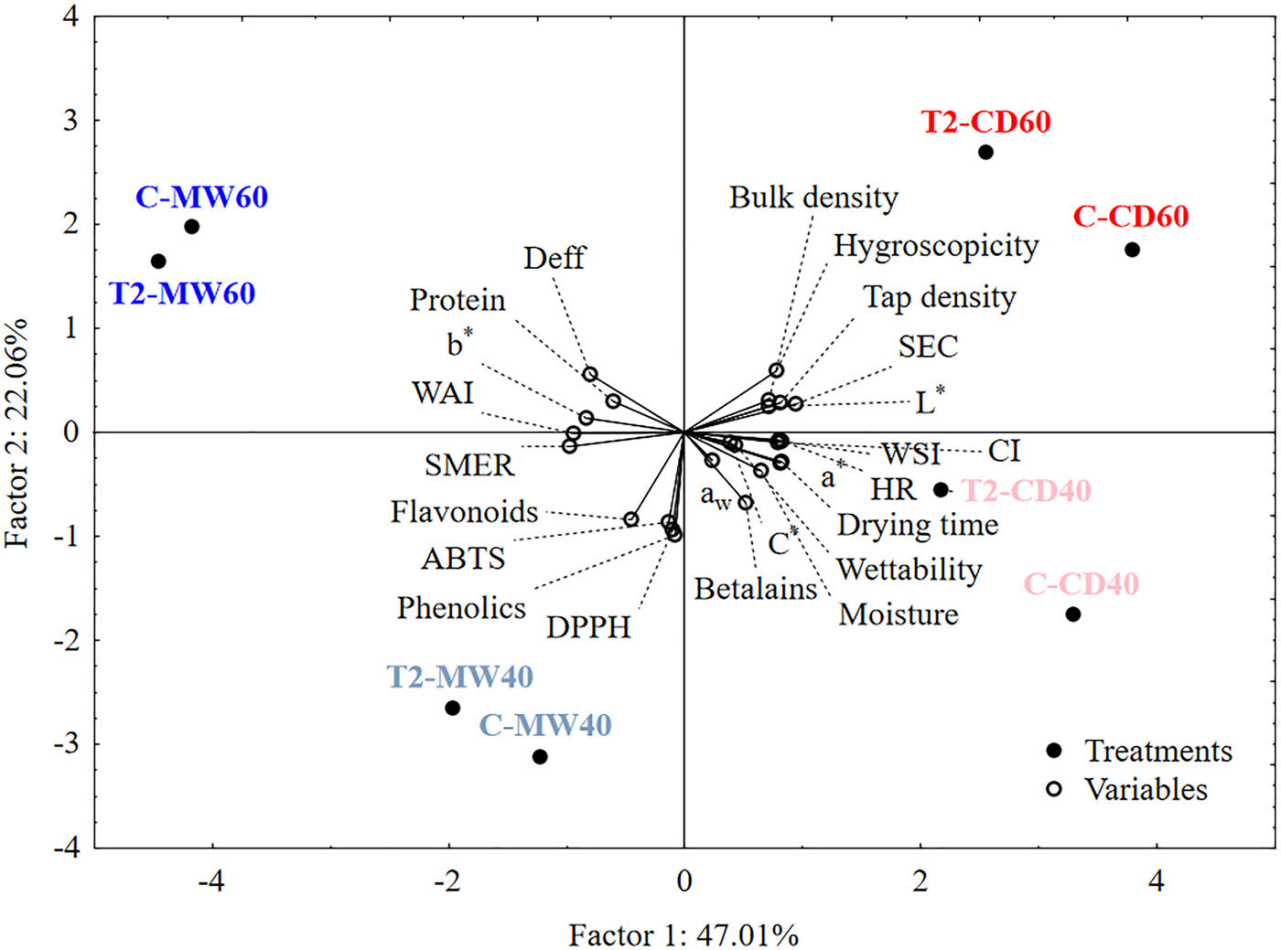
Principal component analysis biplot of pitaya powders obtained under different drying conditions. Vectors represent the contribution and correlation of each variable to the principal components. Variable abbreviations: *D*_eff_ = apparent effective moisture diffusivity; aw = water activity; WSI = water solubility index; WAI = water absorption index; HR = Hausner ratio; CI = Carr index; SEC = specific energy consumption; SMER = specific moisture extraction rate; L*, a*, b*, C*and °h = instrumental color parameters; ABTS and DPPH = antioxidant activity assays. Treatment codes: C = control foam (whey protein); T2 = optimized foam (pea protein); CD = convective drying; MW = microwave drying; 40 and 60 = drying air temperatures (°C).

PCA was applied to elucidate simultaneous correlations between response variables and the different drying treatments, allowing an integrated visualization of the impact of drying method, temperature, and protein source on final pitaya powder quality (Figure 9). The two‐dimensional projection of the first two factors jointly explained 69.07% of total data variance, with 47.01% attributed to Factor 1 and 22.06% to Factor 2, indicating an adequate representation of the multivariate system. The treatment distribution in the biplot showed that sample segregation was predominantly associated with the drying method along Factor 1 and the drying temperature along Factor 2. The complete loading matrix for Factors 1 and 2 is provided in Supplementary Material Table S10, in which variables with absolute loading values equal to or greater than 0.70 are identified as the main contributors to each factor.

The first principal component (Factor 1) discriminated the drying methods, establishing a clear distinction between convective and microwave drying. Convectively dried samples, positioned in the right quadrants, were associated with strong positive loadings for SEC, drying time, bulk density, tapped density, and hygroscopicity. This multivariate pattern is consistent with the longer processing time, higher energy requirement, and denser powder structure observed after CD. In contrast, microwave‐dried samples, located on the negative side of Factor 1, were associated with strong negative loadings for SMER, WAI, and apparent effective moisture diffusivity. The opposite loadings of SEC and SMER clearly reflected the contrasting energy performance of the drying methods, with microwave drying being associated with higher moisture removal per unit of energy and lower specific energy demand under the evaluated laboratory conditions.

Factor 2 primarily reflected the effect of drying temperature, separating samples processed at 60°C, located in the upper quadrants, from those dried at 40°C, positioned in the lower quadrants. The treatments conducted at 40°C were strongly associated with total phenolic content, total flavonoid content, and antioxidant activities determined by DPPH and ABTS, which presented absolute loading values greater than 0.70. Apparent effective moisture diffusivity showed a moderate positive loading on Factor 2, which was consistent with the higher values observed at 60°C in the univariate analysis. Among the treatments conducted at 40°C, the microwave‐dried samples were positioned closer to the phenolic, flavonoid, DPPH, and ABTS vectors. This distribution indicates that microwave drying at 40°C was associated with higher measured contents of selected bioactive compounds and greater antioxidant activity under the evaluated conditions.

Finally, the spatial configuration of the samples showed promising results regarding the use of pea protein as a plant‐based foaming agent. The proximity between pairs of treatments formulated with whey protein and pea protein under the same drying conditions suggests similar behavior for several technological and functional properties of the powders. Both foams responded similarly to the drying conditions evaluated, with the pea‐protein formulation showing comparable performance to whey protein in the formation of the dried matrix and in the preservation of selected bioactive compounds. These results support the potential use of pea protein as a foaming agent for producing value‐added pitaya powders.

## 4 Conclusion

This study showed that foam‐mat drying can be used to obtain pitaya powders with suitable physicochemical properties, bioactive compound retention, and antioxidant activity under the evaluated experimental conditions. The mixture design showed that pea protein outperformed soy protein and albumin among the alternative protein sources evaluated. In addition, no statistically significant differences were detected between the optimized pea protein foam and the whey protein control for the main foam properties, indicating that pea protein showed comparable performance to whey protein under the tested formulation conditions.

Among the evaluated drying methods, intermittent microwave‐assisted drying, especially at 40°C, stood out by significantly reducing process time and energy consumption while promoting greater preservation of bioactive compounds, including phenolics, flavonoids, betalains, and antioxidant activity. In contrast, CD resulted in denser powders with greater water affinity, accompanied by greater structural collapse of the foamed matrix.

Overall, the results indicate that combining pea protein as a foaming agent with intermittent microwave drying at moderate temperature is a promising approach for producing pitaya powder with improved drying performance and preservation of selected bioactive compounds. However, since the experiments were conducted under laboratory‐scale conditions, further studies are needed to evaluate process scale‐up, industrial feasibility, energy performance, and economic viability.

## Author Contributions

**Matheus de Souza Cruz**: Conceptualization, methodology, software, data curation, investigation, formal analysis, project administration, writing – original draft, writing – review and editing. **Amanda Aparecida De Lima Santos**: Writing – review and editing, methodology, investigation, formal analysis. **Gabriela Fonsêca Leal**: Writing – review and editing, formal analysis, methodology, investigation. **Jhenifer Cristina Carvalho Santos**: Methodology, investigation, formal analysis, software, writing – review and editing. **Guilherme Mathias Lopes**: Investigation, formal analysis, writing – review and editing. **Leandro Levate Macedo**: Supervision, validation, conceptualization, writing – review and editing. **Irineu Petri Júnior**: Conceptualization, supervision, writing – review and editing, validation. **Jefferson Luiz Gomes Corrêa**: Writing – review and editing, resources, funding acquisition, supervision, validation.

## Conflicts of Interest

The authors declare no conflicts of interest.

## Supporting information




**Supplementary Table S1‐S10**: jfds71460‐sup‐0001‐TableS1‐S10.docx

## Data Availability

Data available on request from the corresponding author.
