# Supplementary material for: Intermittent Drying With Heated Air and Microwaves of Dragon Fruit Foams Formulated With Different Protein Sources
Source: J Food Sci. 2026 Sep 13;91(9):e71460. doi: 10.1111/1750-3841.71460 (PMC13572783; doi:10.1111/1750-3841.71460)
Supplement: Supplementary file 1 [file JFDS-91-0-s001.docx]

**SUPPLEMENTARY MATERIAL**

**Intermittent drying with heated air and microwaves of dragon fruit foams formulated with different protein sources**

Matheus de Souza Cruz^1^	[matheusnetsouza15@gmail.com](mailto:matheusnetsouza15@gmail.com)	ORCID: 0000-0002-4125-3341

Amanda Aparecida De Lima Santos^1^	[amandalsants@gmail.com](mailto:amandalsants@gmail.com)	ORCID: 0000-0001-8662-4911

Gabriela Fonsêca Leal^1^	[gabrielaleal476@gmail.com](mailto:gabrielaleal476@gmail.com)	ORCID: 0000-0003-1257-2527

Jhenifer Cristina Carvalho Santos^1^	[jhenifercristinacs@gmail.com](mailto:jhenifercristinacs@gmail.com)	ORCID: 0000-0002-9060-3141

Guilherme Mathias Lopes^2^	[gmlopes25@gmail.com](mailto:gmlopes25@gmail.com)	ORCID: 0000-0001-8174-6645

Leandro Levate Macedo^3^	[leandrolevate@hotmail.com](mailto:leandrolevate@hotmail.com)	ORCID: 0000-0003-3010-7485

Irineu Petri Júnior^4^	[irineu.junior@ufla.br](mailto:irineu.junior@ufla.br)	ORCID: 0000-0001-5638-0370

Jefferson Luiz Gomes Corrêa^1*^	[jefferson@ufla.br](mailto:jefferson@ufla.br)	ORCID: 0000-0002-6818-6927

^1^ Department of Food Science, Federal University of Lavras, Lavras, MG, Brazil

^2^ Department of Agricultural Engineering, Federal University of Lavras, Lavras, MG, Brazil

^3^ Faculty of Engineering, Federal University of Grande Dourados, Dourados, MS, Brazil

^4^ Department of Chemical Engineering, Federal University of Lavras, Lavras, MG, Brazil

* Corresponding author: Jefferson Luiz Gomes Corrêa - email: [jefferson@ufla.br](mailto:jefferson@ufla.br) - Address: Trevo Rotatório Professor Edmir Sá Santos, Federal University of Lavras (UFLA), Lavras, MG, 37200-900, Brazil.

**Table S1.** Manufacturer-declared nutritional composition of the commercial protein powders used as foaming agents.

| **Nutritional component** | **Whey protein**  **(NewNutrition)** | **Albumin**  **(NewNutrition)** | **Pea protein**  **(NewNutrition)** | **Soy protein**  **(Empório Terra Vitta)** |
| --- | --- | --- | --- | --- |
| **Energy value (kcal)** | 126 | 107 | 119 | 105 |
| **Carbohydrates (g)** | 2.30 | 0.00 | 1.30 | 0.00 |
| **Total sugars (g)** | NR | NR | 0.00 | NR |
| **Added sugars (g)** | NR | NR | 0.00 | NR |
| **Proteins (g)** | 24.0 | 27.0 | 25.0 | 27.0 |
| **Total fat (g)** | 2.30 | 0.00 | 2.40 | 0.00 |
| **Saturated fat (g)** | 1.00 | 0.00 | NQ | 0.00 |
| **Trans fat (g)** | 0.00 | 0.00 | NQ | NR |
| **Dietary fiber (g)** | 0.00 | 0.00 | NQ | 1.50 |
| **Sodium (mg)** | 39 | 318 | 344 | 300 |

Values correspond to the manufacturer-declared nutritional composition per 30 g serving. NR = not reported; NQ = not quantified because the component was not present in a significant amount according to the product label.

**Table S2.** Minimum, mean and maximum temperatures recorded during intermittent microwave and convective drying of dragon fruit foams.

| **Codes** | **Mean temperature (ºC)** | **Minimum temperature (ºC)** | **Maximum temperature (ºC)** |
| --- | --- | --- | --- |
| **C-CD40** | 42.5±2.3 b | 37.9±1.8 b | 51.2±2.7 b |
| **C-CD60** | 63.7±3.1 a | 56.1±1.1 a | 70.9±3.4 a |
| **T2-CD40** | 43.1±3.6 b | 37.1±1.0 b | 49.1±3.0 b |
| **T2-CD60** | 64.0±2.7 a | 55.9±1.2 a | 69.0±2.3 a |
| **C-MW40** | 42.9±2.7 b | 38.6±1.1 b | 48.0±1.7 b |
| **C-MW60** | 62.7±1.4 a | 58.1±1.1 a | 67.7±1.4 a |
| **T2-MW40** | 43.6±2.5 b | 38.2±1.2 b | 47.6±2.5 b |
| **T2-MW60** | 63.7±1.7 a | 57.4±1.3 a | 68.1±2.2 a |

The values are expressed as the mean of triplicates ± standard deviation (*n* = 3). Different letters in the same column indicate a significant difference between treatments (p < 0.05) according to the Tukey test. Treatment codes: C = control foam (whey protein concentrate); T2 = optimized foam (pea protein); CD = convective drying; MW = microwave drying; 40 and 60 = drying air temperatures (°C).

**Table S3.** Flowability classification based on Carr index (CI) and Hausner ratio (HR) values.

| **Flowability** | **CI (%)** | **HR** |
| --- | --- | --- |
| **Excellent** | 0–10 | 1.00–1.11 |
| **Good** | 11–15 | 1.12–1.18 |
| **Fair** | 16–20 | 1.19–1.25 |
| **Passable** | 21–25 | 1.26–1.34 |
| **Poor** | 26–31 | 1.35–1.45 |
| **Very poor** | 32–37 | 1.46–1.59 |
| **Very, very poor** | > 38 | > 1.60 |

**Table S4.** P-values obtained from factorial ANOVA for the effects of drying method, temperature, foam formulation and their interactions on drying kinetics, energy parameters and model-fitting parameters of pitaya powders.

| **Response variable** | **M** | **T** | **F** | **M × T** | **M × F** | **T × F** | **M × T × F** |
| --- | --- | --- | --- | --- | --- | --- | --- |
| **Drying time (min)** | <0.001* | <0.001* | 1.000ⁿˢ | <0.001* | 0.180ⁿˢ | 0.730ⁿˢ | 0.730ⁿˢ |
| **Duty cycle (%)** | <0.001* | <0.001* | 0.014* | <0.001* | 0.273ⁿˢ | 0.102ⁿˢ | 0.549ⁿˢ |
| **Energy consumption (kWh)** | <0.001* | 0.228ⁿˢ | 0.547ⁿˢ | 0.090ⁿˢ | 0.295ⁿˢ | 0.754ⁿˢ | 0.786ⁿˢ |
| **SMER (kg/kWh)** | <0.001* | 0.047* | 0.254ⁿˢ | 0.036* | 0.218ⁿˢ | 0.932ⁿˢ | 0.912ⁿˢ |
| **SEC (kWh/kg)** | <0.001* | 0.389ⁿˢ | 0.507ⁿˢ | 0.150ⁿˢ | 0.277ⁿˢ | 0.861ⁿˢ | 0.863ⁿˢ |
| **Apparent D*_eff_* x10^8^(m^2^/s)** | <0.001* | <0.001* | 0.263ⁿˢ | <0.001* | 0.316ⁿˢ | 0.812ⁿˢ | 0.921ⁿˢ |
| **R^2^** | <0.001* | 0.694ⁿˢ | 0.148ⁿˢ | 0.338ⁿˢ | 0.407ⁿˢ | 0.532ⁿˢ | 0.755ⁿˢ |
| **RMSE** | <0.001* | 0.156ⁿˢ | 0.646ⁿˢ | 0.064ⁿˢ | 0.387ⁿˢ | 0.913ⁿˢ | 0.520ⁿˢ |
| **χ²** | <0.001* | 0.003* | 0.325ⁿˢ | 0.003* | 0.763ⁿˢ | 0.345ⁿˢ | 0.183ⁿˢ |

Asterisks indicate significant effects at *p* < 0.05. ns indicates non-significant effects. M = drying method; T = drying temperature; F = foam formulation. Variable abbreviations: SMER = specific moisture extraction rate; SEC = specific energy consumption; D_eff_ = effective diffusivity; R² = determination coefficient; RMSE = root mean square error; = reduced chi-square.

**Table S5.** P-values obtained from factorial ANOVA for the effects of drying method, temperature, foam formulation and their interactions on the physicochemical properties of pitaya powders.

| **Response variable** | **M** | **T** | **F** | **M × T** | **M × F** | **T × F** | **M × T × F** |
| --- | --- | --- | --- | --- | --- | --- | --- |
| **Yield (%)** | 0.195^ns^ | 0.071^ns^ | 0.223^ns^ | 0.154^ns^ | 0.837^ns^ | 0.571^ns^ | 0.930^ns^ |
| **ρ_B_ (g/cm^3^)** | <0.001* | 0.989^ns^ | 0.068^ns^ | <0.001* | <0.001* | <0.001* | 0.023* |
| **ρ_T_ (g/cm^3^)** | <0.001* | 0.857^ns^ | 0.058^ns^ | 0.012* | <0.001* | <0.001* | 0.004* |
| **CI** | 0.002* | 0.524^ns^ | 0.965^ns^ | 0.011* | 0.008* | 0.351^ns^ | 0.643^ns^ |
| **HR** | 0.004* | 0.558^ns^ | 0.814^ns^ | 0.018* | 0.014* | 0.483^ns^ | 0.724^ns^ |
| **WSI (%)** | <0.001* | 0.141^ns^ | <0.001* | <0.001* | 0.249^ns^ | 0.029* | 0.520^ns^ |
| **WAI** | <0.001* | 0.127^ns^ | 0.275^ns^ | 0.057^ns^ | 0.047* | 0.785^ns^ | 0.132^ns^ |
| **Hygroscopicity** | <0.001* | <0.001* | 0.393^ns^ | 0.869^ns^ | 0.091^ns^ | 0.770^ns^ | 0.608^ns^ |
| **Wettability (s)** | <0.001* | 0.266^ns^ | <0.001* | 0.064^ns^ | 0.043* | 0.135^ns^ | 0.019* |
| **Moisture (%)** | 0.340^ns^ | 0.380^ns^ | 0.697^ns^ | 0.260^ns^ | 0.459^ns^ | 0.462^ns^ | 0.840^ns^ |
| **a_w_** | 0.566^ns^ | 0.490^ns^ | 0.230^ns^ | 0.698^ns^ | 0.003* | 0.206^ns^ | 0.965^ns^ |

Asterisks indicate significant effects at *p* < 0.05. ns indicates non-significant effects. M = drying method; T = drying temperature; F = foam formulation. Variable abbreviations: ρ_B_ = bulk density; ρ_T_ = tapped density; CI = carr index; HR = hausner ratio; WSI = water solubility index; WAI = water absorption index; aw = water activity.

**Table S6.** Color parameters of the dried pitaya powders

| **Codes** | **L*** | **a*** | **b*** | **C*** | **h°** |
| --- | --- | --- | --- | --- | --- |
| **C-CD40** | 25.62±3.49 ab | 33.22±0.71 a | 2.00±0.87 c | 33.29±0.72 a | 2.73±0.47 e |
| **C-CD60** | 25.33±0.30 ab | 30.33±3.63 abc | 2.58±0.54 c | 30.42±3.65 ab | 4.94±0.87 e |
| **T2-CD40** | 24.87±2.19 ab | 30.73± 1.05 ab | 8.12±1.91 b | 31.81±4.18 ab | 14.67±2.95 d |
| **T2-CD60** | 29.38±0.34 a | 35.30±2.30 a | 1.42±0.86 c | 35.33±2.34 a | 1.53±0.35 e |
| **C-MW40** | 20.23±2.30 bc | 32.15±4.00 a | 8.68±0.45 b | 32.80±3.11 a | 15.43±2.44 d |
| **C-MW60** | 23.04±1.70 bc | 22.88±1.33 c | 11.98±0.32 b | 25.17±1.10 b | 25.17±4.48 c |
| **T2-MW40** | 25.04±1.49 ab | 29.39±4.19 abc | 7.68±2.70 b | 30.57±4.02 ab | 33.43±1.17 b |
| **T2-MW60** | 19.22±1.78 c | 23.64±1.46 bc | 24.27±2.94 a | 33.94±1.99 a | 47.23±4.15 a |

The parameters evaluated include: L* = lightness, a* = red-green axis, b* = yellow-blue axis, C* = chroma and ºh = hue angle. The values are expressed as the mean of triplicates ± standard deviation (*n* = 3). Different letters in the same column indicate a significant difference between treatments (p < 0.05) according to the Tukey test. Treatment codes: C = control foam (whey protein); T2 = optimized foam (pea protein); CD = convective drying; MW = microwave drying; 40 and 60 = drying air temperatures (°C).

**Table S7.** P-values obtained from factorial ANOVA for the effects of drying method, temperature, foam formulation and their interactions on color parameters of pitaya powders.

| **Response variable** | **M** | **T** | **F** | **M × T** | **M × F** | **T × F** | **M × T × F** |
| --- | --- | --- | --- | --- | --- | --- | --- |
| **L*** | <0.001* | 0.710^ns^ | 0.199^ns^ | 0.038* | 0.480^ns^ | 0.250^ns^ | <0.001* |
| **a*** | <0.001* | 0.008* | 0.912^ns^ | 0.002* | 0.321^ns^ | 0.023* | 0.381^ns^ |
| **b*** | <0.001* | <0.001* | <0.001* | <0.001* | 0.032* | 0.040* | <0.001* |
| **C*** | 0.063^ns^ | 0.401^ns^ | 0.030* | 0.258^ns^ | 0.469^ns^ | <0.001* | 0.286^ns^ |
| **h°** | <0.001* | 0.009* | <0.001* | <0.001* | <0.001* | 0.018* | <0.001* |

Asterisks indicate significant effects at *p* < 0.05. ns indicates non-significant effects. M = drying method; T = drying temperature; F = foam formulation. The parameters evaluated include: L* = lightness, a* = red-green axis, b* = yellow-blue axis, C* = chroma and ºh = hue angle.

| **Codes** | **Protein** | **Phenolics** | **Flavonoids** | **Betalains** | **DPPH** | **ABTS** |
| --- | --- | --- | --- | --- | --- | --- |
| **C-CD40** | 26.83±1.01 c | 191.82±6.07 bc | 7.907±0.272 c | 61.880±2.904 a | 56.190±4.479 a | 55.745±4.209 a |
| **C-CD60** | 28.88±3.81 bc | 123.63±3.51 de | 6.929±0.155 d | 46.859±0.900 b | 33.853±3.507 b | 24.147±4.525 c |
| **T2-CD40** | 28.58±2.20 bc | 177.83±7.47 c | 8.245±0.375 c | 42.315±1.406 bc | 56.623±4.935 a | 62.478±1.673 a |
| **T2-CD60** | 33.83±3.54 ab | 110.95±7.20 e | 6.503±0.320 d | 34.169±0.814 d | 29.784±8.740 b | 34.695±3.044 b |
| **C-MW40** | 28.58±1.34 bc | 216.80±6.52 a | 9.856±0.478 b | 61.584±1.723 a | 57.316±3.908 a | 62.118±2.803 a |
| **C-MW60** | 33.83±3.07 ab | 135.43±7.06 d | 7.885±0.097 c | 34.653±0.561 d | 40.606±1.967 b | 41.023±5.638 b |
| **T2-MW40** | 35.88±1.75 a | 206.65±14.04 ab | 10.893±0.364 a | 40.609±2.405 c | 64.242±4.199 a | 59.919±0.897 a |
| **T2-MW60** | 33.10±0.25 abc | 137.04±6.59 d | 8.083±0.135 c | 19.543±0.858 e | 39.827±0.835 b | 40.305±1.088 b |

**Table S8.** Protein content and bioactive compounds of dried pitaya foams obtained under different drying treatments.

The values are expressed as the mean of triplicates ± standard deviation (*n* = 3). Different letters in the same column indicate a significant difference between treatments (p < 0.05) according to the Tukey test. Treatment codes: C = control foam (whey protein); T2 = optimized foam (pea protein); CD = convective drying; MW = microwave drying; 40 and 60 = drying air temperatures (°C). Protein = %; Phenolics = mg GAE/100 g dry basis; Flavonoids = mg/100 g dry basis; Betalains = mg/100 g dry basis; DPPH and ABTS = %FRS.

**Table S9.** P-values obtained from factorial ANOVA for the effects of drying method, temperature, foam formulation and their interactions on protein content, bioactive compounds and antioxidant activity of pitaya powders.

| **Response variable** | **M** | **T** | **F** | **M × T** | **M × F** | **T × F** | **M × T × F** |
| --- | --- | --- | --- | --- | --- | --- | --- |
| **Protein** | 0.004* | 0.026* | 0.004* | 0.242^ns^ | 0.970^ns^ | 0.242^ns^ | 0.012* |
| **Phenolics** | <0.001* | <0.001* | 0.014* | 0.231^ns^ | 0.175^ns^ | 0.321^ns^ | 0.425^ns^ |
| **Flavonoids** | <0.001* | <0.001* | 0.033* | <0.001* | 0.016* | 0.005* | 0.879ns |
| **Betalains** | <0.001* | <0.001* | <0.001* | <0.001* | 0.174^ns^ | <0.001* | 0.713^ns^ |
| **DPPH** | 0.004* | <0.001* | 0.743^ns^ | 0.301^ns^ | 0.213^ns^ | 0.125^ns^ | 0.677^ns^ |
| **ABTS** | <0.001* | <0.001* | 0.020* | 0.004* | 0.002* | 0.353^ns^ | 0.679^ns^ |

Asterisks indicate significant effects at *p* < 0.05. ns indicates non-significant effects. M = drying method; T = drying temperature; F = foam formulation. Protein = %; Phenolics = mg GAE/100 g dry basis; Flavonoids = mg/100 g dry basis; Betalains = mg/100 g dry basis; DPPH and ABTS = %FRS.

**Table S10.** Variable loadings on Factors 1 and 2 obtained from the principal component analysis of pitaya powders produced under different drying conditions.

| **Variable** | **Factor 1** | **Factor 2** | **Variable** | **Factor 1** | **Factor 2** |
| --- | --- | --- | --- | --- | --- |
| Deff | **−0.796** | 0.556 | CI | **0.791** | −0.096 |
| Drying time | **0.820** | −0.285 | HR | **0.799** | −0.070 |
| SEC | **0.940** | 0.273 | Wettability | 0.430 | −0.118 |
| SMER | **−0.975** | −0.137 | Hygroscopicity | **0.777** | 0.589 |
| Moisture | 0.644 | −0.366 | WSI | **0.824** | −0.087 |
| Aw | 0.238 | −0.271 | WAI | **−0.937** | −0.013 |
| L* | **0.709** | 0.245 | Protein | −0.608 | 0.302 |
| a* | **0.811** | −0.294 | Phenolics | −0.079 | **−0.992** |
| b* | **−0.833** | 0.138 | DPPH | −0.101 | **−0.939** |
| C* | 0.384 | −0.098 | ABTS | −0.131 | **−0.864** |
| °h | **−0.901** | −0.034 | Betalains | 0.520 | −0.682 |
| Bulk density | **0.717** | 0.311 | Flavonoids | −0.455 | **−0.835** |
| Tapped density | **0.816** | 0.291 |  |  |  |

Vectors represent the contribution and correlation of each variable to the principal components. Values in bold indicate the main contributors to each factor, defined as absolute loading values ≥ 0.70. Variable abbreviations: Deff = apparent effective moisture diffusivity; aw = water activity; WSI = water solubility index; WAI = water absorption index; HR = Hausner ratio; CI = Carr index; SEC = specific energy consumption; SMER = specific moisture extraction rate; L*, a*, b*, C*and ºh = instrumental color parameters; ABTS and DPPH = antioxidant activity assays. Treatment codes: C = control foam (whey protein); T2 = optimized foam (pea protein); CD = convective drying; MW = microwave drying; 40 and 60 = drying air temperatures (°C).
